# *Solanum bulbocastanum* nucleotide‐binding leucine‐rich repeat receptor evolution reveals functional variants and critical residues in Rpi‐blb1/RB

**DOI:** 10.1111/jipb.13950

**Published:** 2025-06-17

**Authors:** Jie Li, Sophie Mantelin, Miles Armstrong, Amanpreet Kaur, Sonia Gomez, Jiahan Ying, Xiuli Qin, Kathryn M. Wright, Brian Harrower, Paolo Ribeca, Théo Chaumet, Gaynor McKenzie, Huanting Liu, Malcolm F. White, Thomas Adams, Stuart Ronan Fisher, Daolong Dou, Xiaodan Wang, Ingo Hein

**Affiliations:** ^1^ State Key Laboratory of Agricultural and Forestry Biosecurity, MOA Key Lab of Pest Monitoring and Green Management, College of Plant Protection China Agricultural University Beijing 100193 China; ^2^ James Hutton Institute, Invergowrie Dundee DD25DA UK; ^3^ University of Dundee Dundee DD14HN UK; ^4^ National University of Colombia Medellín 111321 Colombia; ^5^ School of Biology University of St Andrews St Andrews KY16 9ST UK; ^6^ College of Plant Protection Nanjing Agricultural University Nanjing 210095 China

**Keywords:** enrichment sequencing, host–pathogen co‐evolution, nucleotide‐binding leucine‐rich repeat (NLR) gene, plant immunity, *Solanum bulbocastanum*

## Abstract

Host–pathogen co‐evolution shapes resistance (*R*) proteins and their recognition of pathogen avirulence factors. However, little attention has been paid to naturally occurring genetic diversity in *R* genes. In this study, 12 *Solanum bulbocastanum* accessions from the Commonwealth Potato Collection were screened for resistance to *Phytophthora infestans*, identifying 11 resistant and one susceptible accession. Targeted enrichment sequencing of nucleotide‐binding leucine‐rich repeat (NLR) genes using RenSeq, followed by diagnostic RenSeq (dRenSeq) analysis, revealed that all accessions except 7650 contained *Rpi‐blb1/RB* variants. Variants in accessions 7641 and 7648 were non‐functional, while three novel functional variants were identified. Cloning and functional analysis of *Rpi‐blb1/RB* variants assessed their recognition of the avirulence factor IPI‐O1. Three variants were functional, conferring resistance to *P. infestans*. Variants in accessions 7644 and 7647 also recognized IPI‐O4, confirmed in transgenic potatoes. Analysis of a non‐functional variant in *S. bulbocastanum* accession 7648 identified amino acid Ser347 in the nucleotide‐binding (NB‐ARC) domain as critical for cell‐death initiation following IPI‐O1 recognition. Predictions from the FunFOLD2 protein–ligand interaction model suggested that Ser347 is essential for ATP binding, suggesting potential inhibition on pentameric resistosome assembly. Western blot analysis revealed that the mutation of Ser347 to Asn markedly compromises the Rpi‐blb1/RB protein stability, and co‐immunoprecipitation assay further confirmed that this mutation severely disrupts the self‐association of CCNB, thereby preventing Rpi‐blb1/RB activation. Consistently, substituting Asn347 with serine restored function, underscoring its key role in Rpi‐blb1/RB activity. Cell biology experiments demonstrated that Rpi‐blb1/RB relocalizes to the plasma membrane in response to IPI‐O1. This relocalization depends on Ser347, further supporting the idea that its mutation affects resistosome formation, impairing resistance. This study provides an in‐depth functional analysis of natural *Rpi‐blb1/RB* diversity, offering insights into NLR protein evolution and resistance mechanisms in potatoes.

## Graphical abstract

Natural variants of the nucleotide‐binding leucine‐rich repeat protein Rpi‐blb1/RB in Solanum bulbocastanum recognize the Phytophthora infestans effector IPI‐O4, as well as two non‐functional variants. The S347N mutation disrupts IPI‐O1 recognition, Rpi‐blb1/RB self‐association and subcellular relocalisation, thereby compromising resistance.

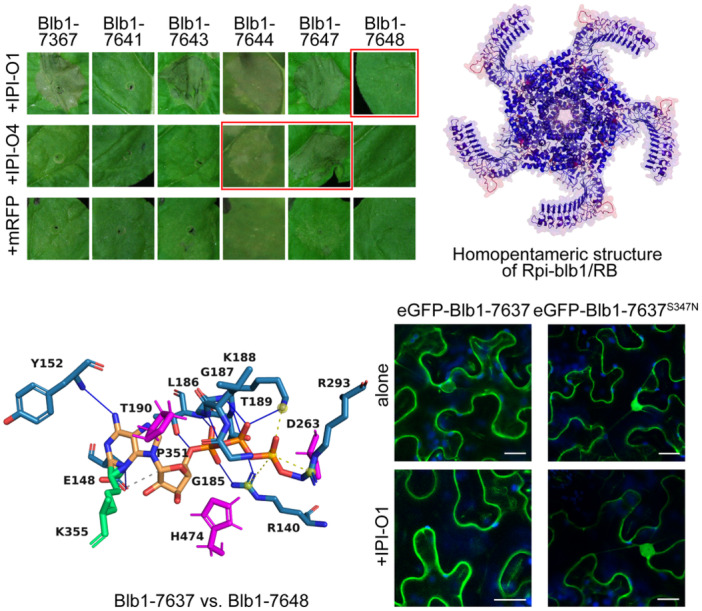

## INTRODUCTION

A key strategy to combat late blight disease in potatoes is the introduction of broad‐spectrum resistance (*R*) genes against *Phytophthora infestans*. This relies on identifying natural resistances in wild potato species and incorporating these into cultivated varieties. Over the past 30 years, many resistances derived from *Solanum demissum* have been mapped to potato chromosomes, with six cloned, including *R1*, *R2*, *R3a*, *R3b*, *R8*, and *R9a* (Paluchowska et al., 2022). However, many of these resistances have been compromised by the emergence of virulent pathogen strains. Consequently, the search has expanded to other wild species, including *Solanum bulbocastanum*, a diploid wild potato known for its late blight resistance. Four resistance genes, *Rpi‐blb1/RB* (Song et al., 2003; Van Der Vossen et al., 2003), *Rpi‐bt1* (Oosumi et al., 2009), *Rpi‐blb2* (Van Der Vossen et al., 2005), and *Rpi‐blb3* (Lokossou et al., 2009), have been cloned, with a novel gene, *Rpi‐blb4*, recently mapped (Li et al., 2023). To date, all cloned potato resistance genes effective against late blight are members of the intracellular nucleotide‐binding leucine‐rich repeat (NLR) family of receptors that detect pathogen effectors.

Plant–pathogen co‐evolution influences the diversity of NLR genes and the cognate pathogen genes they recognize. Nucleotide‐binding leucine‐rich repeats are among the most diverse protein families in angiosperms, with genomes encoding up to 1,000 NLRs (Adachi et al., 2019). In potato, approximately 750 NLRs have been identified in the doubled monoploid clone DM1‐3 516 R44, derived from *Solanum tuberosum* Group Phureja (Jupe et al., 2013), and in tetraploid cultivars such as Innovator, more than 2,400 NLRs have been predicted (Wang et al., 2023). NLR evolution is shaped by cycles of gene duplication, loss, and diversification, leading to extensive sequence divergence (Michelmore and Meyers, 1998). Due to frequent lineage‐specific expansions, reconstructing conserved NLR evolution is challenging (Adachi et al., 2023). Phylogenetic relationships are further obscured by rapid diversification at the intraspecific level (Prigozhin and Krasileva, 2021), limiting mechanistic insights into NLR evolution against dynamic pathogen effectors.

NLR proteins typically contain an N‐terminal domain, such as a coiled‐coil (CC) or a Toll‐interleukin receptor (TIR) domain, a central nucleotide‐binding (NB‐ARC) domain controlling activation, and a C‐terminal leucine‐rich repeat (LRR) domain for effector recognition. Subgroups include CC, CC_R_/RPW8, TIR, and CC_G10_ domains (Förderer and Kourelis, 2023). Recent evidence shows that AtZAR1 and TmSr35 assemble into pentameric resistosomes (Wang et al., 2019; Förderer et al., 2022), inserting into membranes to activate immune responses (Bi et al., 2021; Förderer et al., 2022). NLR evolution has led to functionally specialized receptor pairs and networks, such as NRC4, which is required for Rpi‐blb2, Mi‐1.2, and R1 function (Wu et al., 2017). More recently, NbNRC2 was found to assemble into a homohexameric resistosome in response to Rx and PVX CP (Madhuprakash et al., 2024), while TIR‐containing NLRs (TNLs), such as RPP1 (Ma et al., 2020), form tetrameric resistosomes, broadening the understanding of NLR structural diversity.

The *Rpi‐blb1/RB* gene encodes a CCNB‐LRR (CNL) immune receptor that provides broad‐spectrum resistance to *P. infestans* (Sun et al., 2024). Conserved homologs of Rpi‐blb1/RB have been identified in *Solanum stoloniferum*, including *Rpi‐sto1, Rpi‐plt1, Rpi‐pta1*, and *Rpi‐pta2* (Wang et al., 2008). These genes, originally discovered in species once classified separately as *S. polytrichon* and *S. papita*, are now recognized within the unified species *S. stoloniferum*. *Rpi‐blb1/RB* and homologs recognize the RXLR‐containing effector IPI‐O (*in‐planta*‐induced O) to activate immune responses (Vleeshouwers et al., 2008; Champouret et al., 2009; Xie et al., 2015). IPI‐O effectors are grouped into three classes (Champouret et al., 2009; Halterman et al., 2010). Class I and II effectors (e.g., IPI‐O1, IPI‐O2, and IPI‐O3) trigger cell death upon recognition by Rpi‐blb1/RB, while Class III (e.g., IPI‐O4) suppresses Rpi‐blb1/RB‐mediated immunity (Champouret et al., 2009; Halterman et al., 2010; Chen and Halterman, 2011; Chen et al., 2012). Both IPI‐O1 and Rpi‐blb1/RB are localized in the cytoplasm and nucleus, a distribution essential for triggering hypersensitive cell death and immune responses (Zhao and Song, 2021). Upon recognition of IPI‐O1, Rpi‐blb1 undergoes conformational changes, activating defense signaling (Chen et al., 2012). Interestingly, IPI‐O1 interacts with the potato splicing factor StCWC15, altering its localization from the nucleoplasm to the nucleolus, leading to alternative splicing of Rpi‐blb1/RB and activating defense responses (Sun et al., 2024).

The widespread presence of IPI‐O Class I and II effectors in *P. infestans* ensures that *Rpi‐blb1/RB* remains agronomically relevant (Champouret et al., 2009; Halterman et al., 2010; Karki et al., 2021b). This study provides functional insights into *Rpi‐blb1/RB* diversity within *S. bulbocastanum*, identifying new functional variants capable of recognizing IPI‐O4, suggesting positive selection. A homolog in a susceptible *S. bulbocastanum* accession carries four amino acid differences that impair function, with Ser347 identified as critical for activity. The S347N mutation disrupts IPI‐O1 recognition, CCNB self‐association, and subcellular re‐localization, thereby compromising resistance. These findings reveal the evolutionary dynamics shaping *Rpi‐blb1/RB* adaptation, where functional paralogues drive immune responses, while pseudogenized variants potentially reflect past host–pathogen interactions.

## RESULTS

### Phenotypic assessment of 12 *S. bulbocastanum* accessions for resistance against *P. infestans* identifies only one susceptible accession

**Figure 1:**
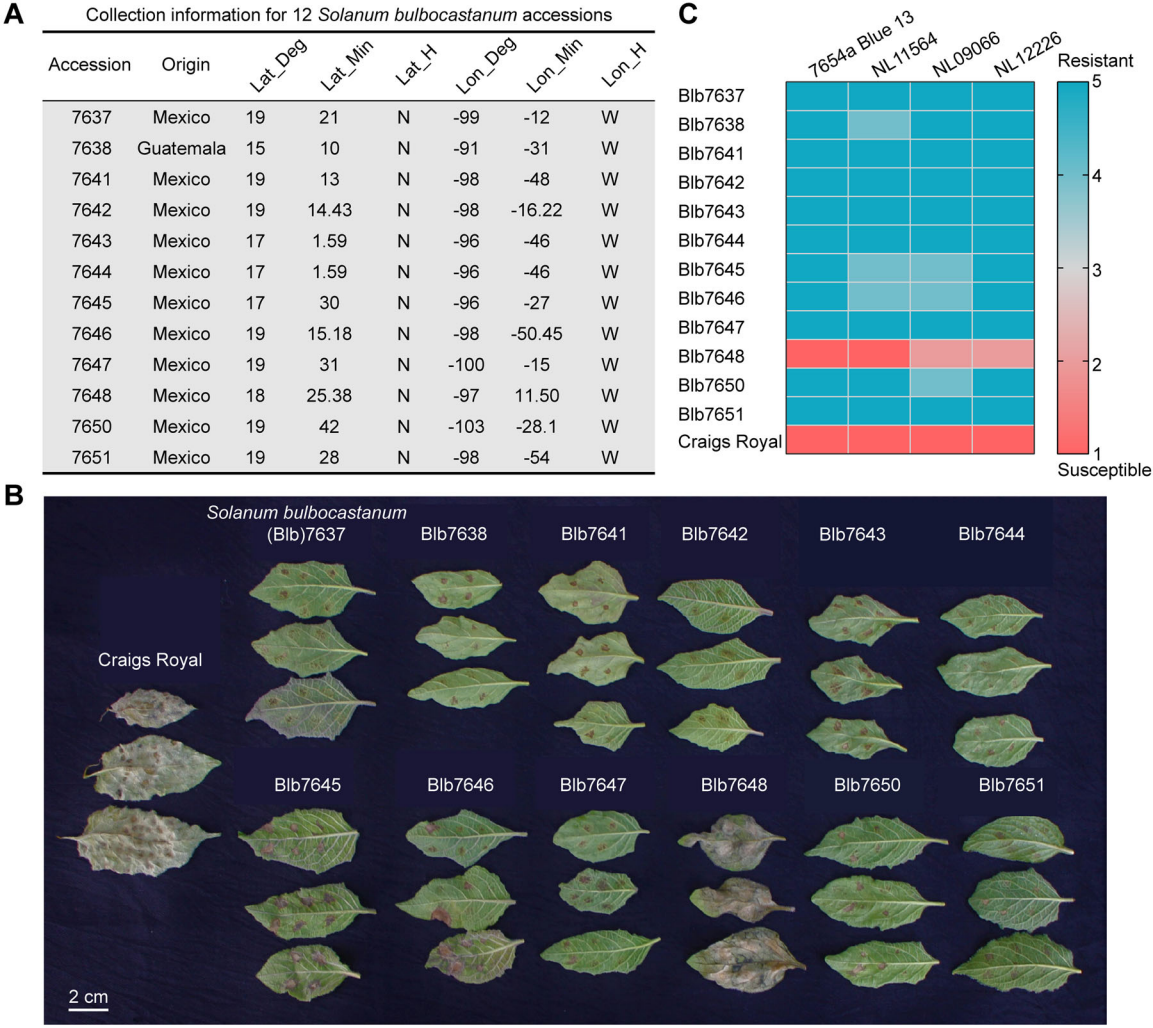
**Distribution and late blight phenotype of 12 accessions of** ***Solanum bulbocastanum*** **(A)** Collection information for 12 accessions of *S. bulbocastanum* from the Commonwealth Potato Collection (CPC). **(B)** Late blight phenotype of 12 accessions of *S. bulbocastanum* to *Phytophthora infestans* isolate 7654a Blue 13 alongside cv. Craigs Royal as a susceptible control. **(C)** Heat map of disease severity of 12 accessions and the susceptible control Craigs Royal to different *P. infestans* isolates. Disease severity (late blight phenotype) was recorded on the Malcolmson scale where 1 refers to highly susceptible (red) and 5 refers to highly resistant (blue).

A selection of 12 *S. bulbocastanum* accessions from the Commonwealth Potato Collection (CPC), was assessed for resistance to the aggressive late blight isolate 2009‐7654A, belonging to the genotype 13_A2 (Cooke et al., 2012). The accessions originated from diverse locations in Mexico and Guatemala (Figure 1A). The accessions: 7637, 7638, 7641, 7642, 7643, 7644, 7645, 7646, 7647, 7650, and 7651 displayed strong resistance to *P. infestans*, whereas accession 7648 was found to be highly susceptible (Figure 1B). Based on a 1–5 score in detached‐leaf assays, where 1 is highly susceptible and 5 highly resistant, the 11 resistant accessions yielded a score of 5, whereas 7648 scored 1. Additional disease assays with late blight isolates NL11564, NL09066 and NL12226 yielded very similar results with scores ranging from 4 to 5 for the resistant accessions and 1 to 2 for 7648 (Figure 1C).

### dRenSeq analysis identifies functional NLRs and novel variants of *Rpi‐blb1/RB*

**Figure 2:**
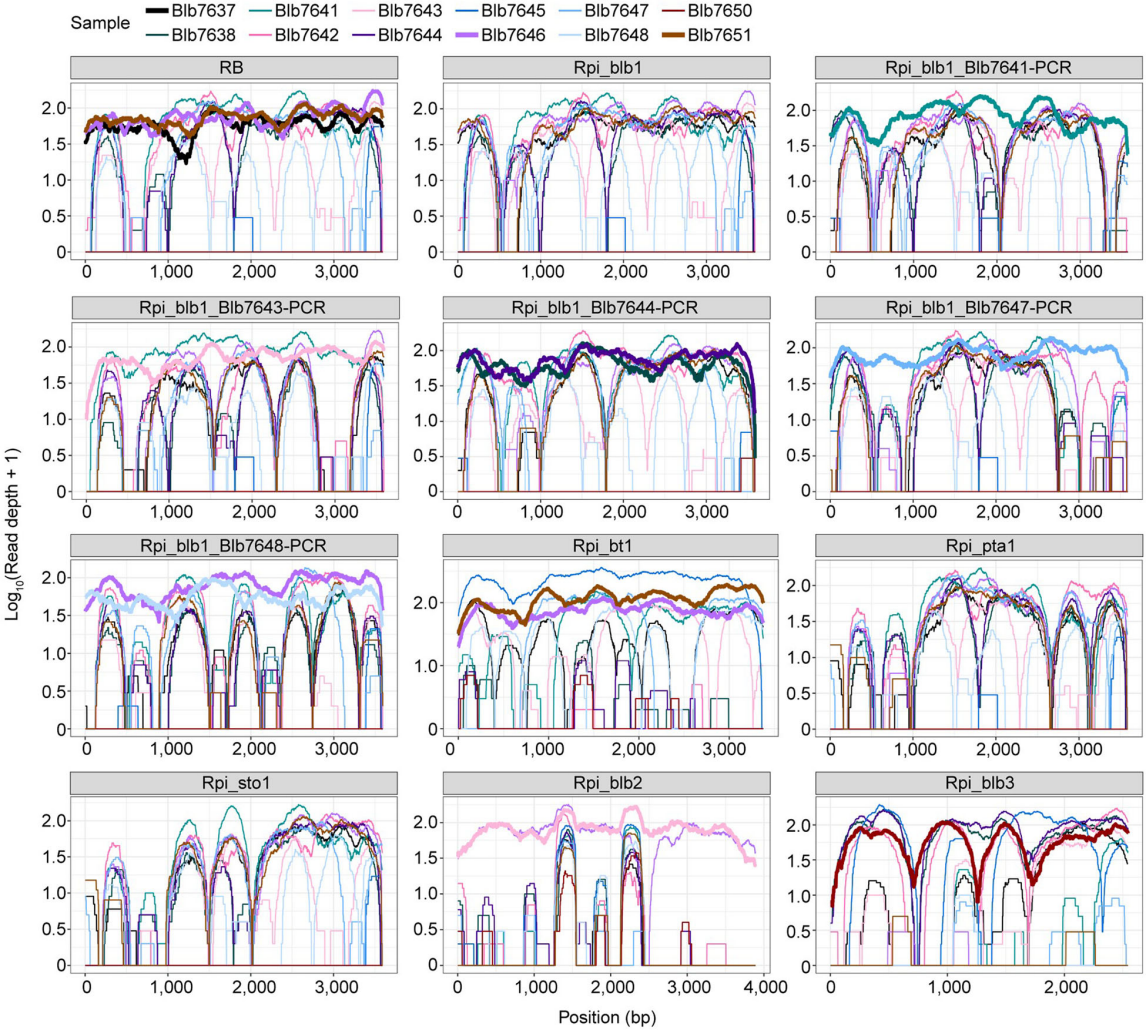
**dRenSeq analysis identifies functional nucleotide‐binding leucine‐rich repeats (NLRs) and novel variants of** ***Rpi‐blb1/RB*** dRenSeq analysis of 12 *Solanum bulbocastanum* accessions. The RenSeq‐generated sequencing reads undergo rigorous alignment with established *NLR* gene references under stringent parameters. In the graphical representation, the *x*‐axis delineates the complete coding span of each NLR locus, whereas the *y*‐axis employs logarithmic scaling to quantify NB‐LRR‐specific coverage depth.

Four known functional disease resistance genes effective against late blight (*Rpi‐blb1*, *Rpi‐blb2*, *Rpi‐blb3*, and *Rpi‐bt1*) have been characterized in *S. bulbocastanum* previously (Van Der Vossen et al., 2003, 2005; Lokossou et al., 2009; Oosumi et al., 2009). We used dRenSeq (Armstrong et al., 2019) to assess their presence in all 12 accessions (Figure 2; Table S1). Also included in the dRenSeq reference set were naturally occurring variants of *Rpi‐blb1* such as *RB* (Song et al., 2003), *Rpi‐sto1* from *S. stoloniferum* (Vleeshouwers et al., 2008) and, *Rpi‐pta1* from *S. papita* (Vleeshouwers et al., 2008). The proteins encoded by *Rpi‐blb1* and *RB* are 100% identical as the genes are only distinguishable by three single nucleotide polymorphisms (SNPs) in the intron sequence.

The dRenSeq analysis suggested the presence of sequences identical to the references *RB* (accessions 7637, 7646, and 7651), *Rpi‐blb2* (accession 7643), *Rpi‐blb3* (accession 7650) and *Rpi‐bt1* (accessions 7646 and 7651). Accession 7646 contained, in addition to functional *RB* and *Rpi‐bt1*, RenSeq reads that covered 99% of the *Rpi‐blb2* reference. Similarly, accession 7645 contained reads that mapped to 99.4% of the *Rpi‐bt1* reference. These accessions presumably contain sequences very similar to the reference sequences but with polymorphisms that prevent full coverage. The highest frequency of naturally occurring sequence variation was identified for *Rpi‐blb1/RB*. In addition to the three accessions with identical coding sequences to *RB*, eight of the 12 accessions contained polymorphic *Rpi‐blb1/RB* sequences (7638, 7641, 7642, 7643, 7644, 7646, 7647, and 7648) (Table 1).

**Table 1:** Percentage coverage of selected functional nucleotide‐binding leucine‐rich repeat (NLR) genes with RenSeq reads from 12 *Solanum bulbocastanum* accessions

	7637	7638	7641	7642	7643	7644	7645	7646	7647	7648	7650	7651
*RB*	100.0	94.5	95.6	98.5	88.0	90.3	12.3	100.0	86.8	78.1	0.0	100.0
*Rpi_blb1*	92.8	96.7	97.5	99.4	86.1	96.3	12.3	98.8	93.7	80.7	0.0	93.4
*Rpi_blb1_Blb7637*	100.0	90.2	95.6	98.5	83.7	89.6	5.7	100.0	87.5	78.4	0.0	100.0
*Rpi_blb1_Blb7641*	89.3	97.9	100.0	95.3	83.2	90.3	11.0	94.6	90.9	82.5	0.0	88.2
*Rpi_blb1_Blb7643*	86.5	85.6	98.9	90.7	100.0	85.7	12.5	79.0	81.8	74.1	0.0	78.6
*Rpi_blb1_Blb7644*	91.4	100.0	98.3	95.3	92.5	100.0	8.2	95.5	89.4	78.5	4.8	90.5
*Rpi_blb1_Blb7647*	80.0	89.0	86.0	93.7	77.8	88.7	15.5	89.0	100.0	80.1	0.0	75.9
*Rpi_blb1_Blb7648*	87.0	83.7	90.3	89.9	76.4	84.9	18.5	100.0	80.9	100.0	0.0	78.1
*Rpi_bt1*	90.2	47.3	94.4	12.5	92.6	34.6	99.4	100.0	69.9	90.0	20.8	100.0
*Rpi_pta1*	93.8	88.0	89.5	90.5	80.1	89.1	5.4	86.8	82.2	72.8	0.0	91.0
*Rpi_sto1*	86.1	85.4	89.7	85.3	74.0	84.8	5.4	78.8	85.1	69.6	0.0	83.9
*Rpi_blb2*	25.8	31.3	35.5	42.3	100.0	47.9	27.1	99.0	20.7	34.9	32.4	25.4
*Rpi_blb3*	72.1	98.2	35.1	93.0	69.0	98.2	81.0	15.2	31.2	8.7	100.0	17.1

*Note*: All values are percentages, and complete coverage is highlighted in red.

In order to determine the nature of the *Rpi‐blb1/RB* variants present in these accessions, we relaxed the stringency of the read mapping until 100% coverage of the reference sequence was achieved and deduced the sequences of the variants by examining the alignments between the RenSeq reads and the published references. This approach, which confirmed sequence polymorphisms in functional NLRs previously (Armstrong et al., 2019), successfully predicted the Rpi‐blb1/RB sequence polymorphisms in 7638, 7641, 7643, 7644, 7647, and 7648 (Figure S1; Table S2) but could not discern the complex haplotypes present in accession 7642. In total, we identified 63 sequence polymorphisms between all accessions for *Rpi‐blb1/RB*‐like sequences. Based on the resulting unique coding sequence, five distinct protein variants were predicted that we referred to as Blb1‐7641, Blb1‐7643, Blb1‐7644, Blb1‐7647, and Blb1‐7648. The variant Blb1‐7644 was also independently identified in accessions 7638 as was the variant Blb1‐7648 in accession 7646 (Table 1).

To confirm the dRenSeq‐derived sequence predictions for *Rpi‐blb1/RB* and its variants in *S. bulbocastanum*, the genes were PCR amplified from genomic DNA. Consistent with the dRenSeq analysis, all accessions yielded a PCR product of the expected size for *Rpi‐blb1/RB*, apart from 7642 that did not produce any amplicon. Full‐length *Rpi‐blb1/RB*‐like sequences were subsequently cloned into a Gateway entry vector and recombinant plasmids sequenced. The sequence analysis confirmed all polymorphisms deduced in the dRenSeq analysis and did not identify further variations, highlighting both the accuracy and the efficacy of the approach. The sequencing of the *Rpi‐blb1/RB* clone from the accession 7641 confirmed the presence of a C/G SNP at position 2,040 that introduces a premature stop codon (codon 454) in the sequence (Table S2), which should result in a truncated protein. This mutation was observed previously in the susceptible allele of the *RB* gene (Song et al., 2003). Remarkably, the accession 7648 that is susceptible to late blight (Figure 1B, C) encodes an *Rpi‐blb1/RB* variant with 13 SNPs of which only four are non‐synonymous (Table S2).

### RenSeq *k‐*mer analysis reveals NLR diversity independent of geographical origin

**Figure 3:**
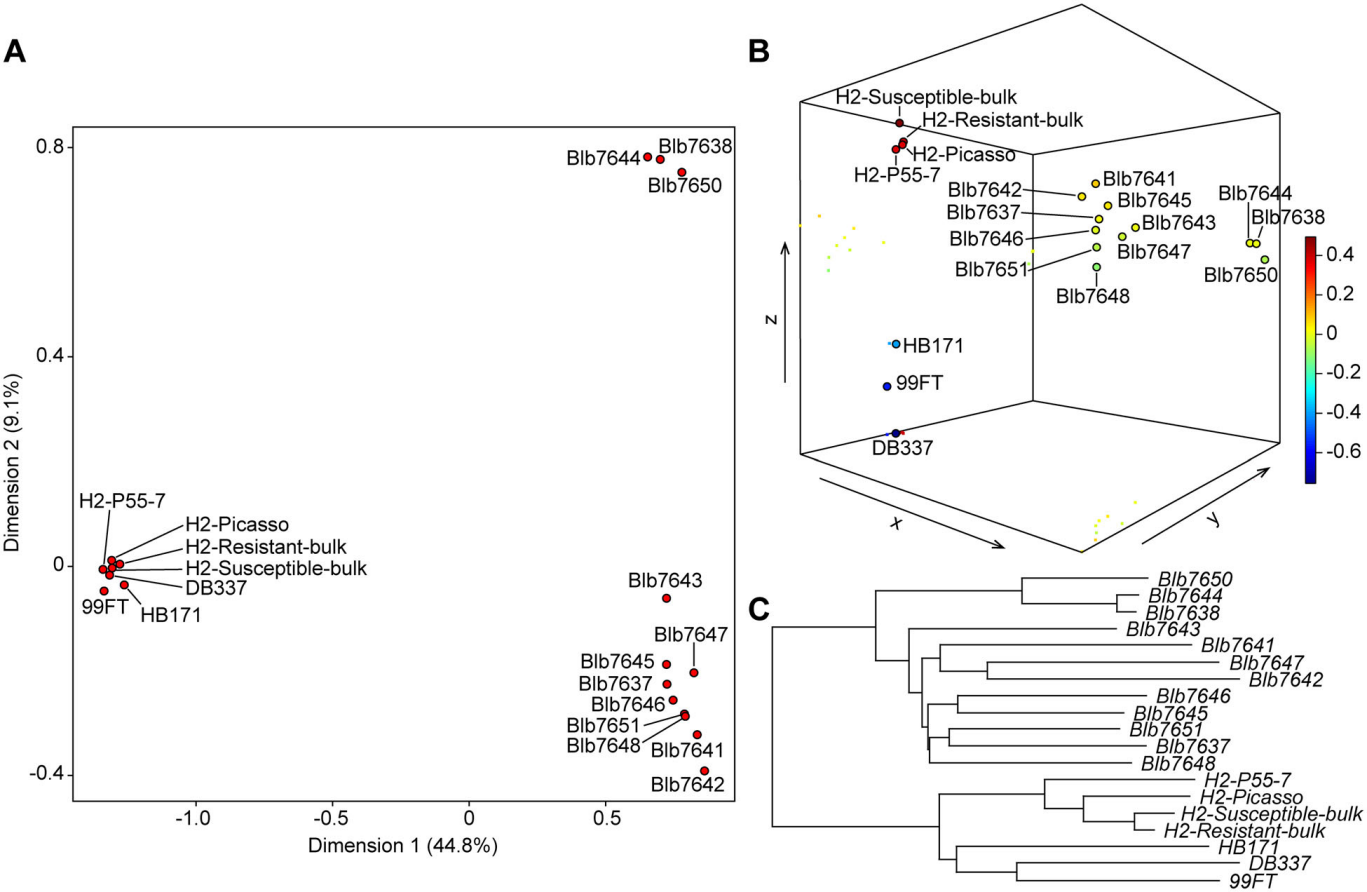
Three equivalent representations of the results of the KPop *k*‐mer analysis of unassembled RenSeq reads **(A−C)** Plots showing the first two dimensions of the positions of the datasets in KPop twisted space **(A)**, first three dimensions **(B)**, and a pseudo‐phylogenetic tree generated from the distances in KPop twisted space **(C)**.

To appraise the global diversity of NLRs within the *S. bulbocastanum* accessions, RenSeq data were used for *k‐*mer analysis in which a genetic distance between samples is estimated based on the spectra of *k‐*mers present in the samples. In addition to the 12 *S. bulbocastanum* accessions, seven accessions from *S. tuberosum* and the *S. tuberosum* Group Phureja were included (Table S1) to test the sensitivity of the method. These accessions included parents and grandparents of the population 06H1 (HB171(13), 99FT.1b5 and its parent DB337(73)) used previously to map the PVY resistance Ry(o)phu (Torrance et al., 2020). Further, we included the breeding clone P55/7 and the cultivar Picasso alongside the two F1 bulks H2‐R and H2‐S used to map the *H2* nematode resistance gene (Strachan et al., 2019). The *k‐*mer analysis clearly distinguished the RenSeq‐derived reads from *S. tuberosum/S. tuberosum* Group Phureja from the *S. bulbocastanum* accessions and divided the latter into two groups (Figure 3). Interestingly, the smaller cluster comprising accessions 7650, 7644, and 7638 represents plants collected from Northern and Southern Mexico as well as Guatemala, respectively. Further, the analysis distinguished accessions that were collected from proximal locations such as 7643 and 7644 (collected independently at the Ruins of Monte Alban in the state of Oaxaca (Figure 1A)) indicating that the NLR diversity in these accessions of *S. bulbocastanum* is not a feature of their geographical origin.

### Functional analysis of *Rpi‐blb1/RB* variants in reducing *P. infestans* lesion size

**Figure 4:**
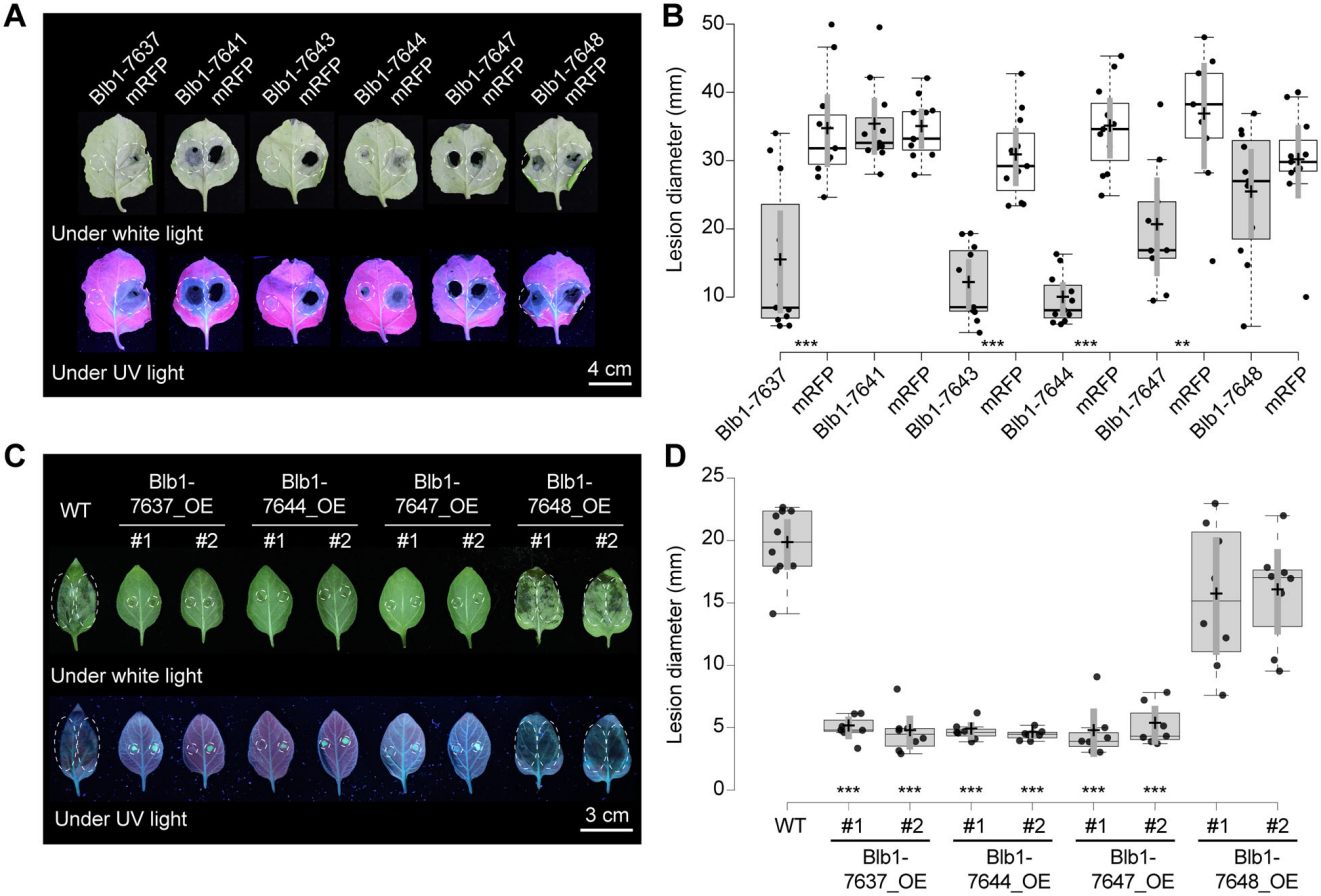
**Effect of Rpi‐blb1/RB variants on** ***Phytophthora infestans*** **lesion size** **(A)** *Phytophthora infestans* infection of *Nicotiana benthamiana* leaves following the transient *Agrobacterium*‐mediated overexpression of *Rpi‐blb1/RB* variants or *mRFP*. Symptoms were recorded 13 d post‐inoculation. Pictures were taken under white light and UV light. **(B)** Statistical analysis of the results obtained in **(A)** for *n* = 12 sample points. **(C)** Effect of key *Rpi‐blb1/RB* variants on *P. infestans* lesion size in stable transgenic potatoes. *Phytophthora infestans* infection of wild‐type or stable transgenic potatoes overexpressing *Rpi‐blb1/RB* variants, recorded 5 d post‐inoculation. Pictures were taken under white light and UV light. **(D)** Statistical analysis of the results obtained in **(C)** for *n* = 10, 7, 8, 7, 8, 7, 7, 8, 8 sample points, respectively. In **(B**, **D)**, center lines show the medians; box limits indicate the 25th and 75th percentiles as determined by R software; whiskers extend 1.5 times the interquartile range from the 25th and 75th percentiles, outliers are represented by dots; crosses represent sample means; bars indicate 95% confidence intervals of the means; data points are plotted as open circles. Significantly different means were calculated using two‐sample *t*‐tests assuming equal variances and are indicated by asterisks, **P < 0.01; ***P < 0.001.

Of the 12 *S. bulbocastanum* accessions tested, 11 were resistant to *P. infestans* (Figure 1B, C). The presence of an originally described *Rpi‐blb1/RB* sequence in accessions 7637, 7646, and 7651, as well as *Rpi‐blb2* in 7643 (Figure 2; Table 1) can explain the observed resistance phenotype in these accessions as late blight isolates of the 13_A2 genotype are known to be avirulent on *Rpi‐blb1/RB* or *Rpi‐blb2* containing hosts (Cooke et al., 2012). However, the resistance of the remaining seven accessions cannot be so easily explained. One possibility is that naturally occurring variants of *Rpi‐blb1/RB* are responsible for the observed resistance. To test this hypothesis, we transiently expressed the cloned wild‐type (WT) *Rpi‐blb1/RB* from 7637 and its variants from 7643, 7644, and 7647 in *Nicotiana benthamiana*. The leaves were subsequently inoculated with *P. infestans*, and the development of the resulting lesions recorded to assess the functionality of the NLR variants (Figures 4A, B, S2). As N‐terminal tagging of Rpi‐blb1/RB compromises recognition of its cognate effector IPI‐O1 (Figure S3; Table S3), the untagged version of the proteins was used. In addition, the variants from 7641 (coding for truncated Rpi‐blb1/RB) and the variant from the susceptible accession 7648 were used as negative controls. As expected, neither the truncated variant from 7641 nor the variant from the susceptible accession 7648 conferred resistance to *P. infestans*, with lesions that developed as much as on the mRFP counterpart (Figure 4A, B). By contrast, expression of the original *Rpi‐blb1/RB* form from 7637 led to a significant reduction of the blight development. Similarly, expression of the *Rpi‐blb1/RB* variants from 7643, 7644, and 7647 also significantly compromised *P. infestans* growth. Therefore, we concluded that the *Rpi‐blb1/RB* variants present in *S. bulbocastanum* accessions 7643, 7644, and 7647 encoded functional NLRs, while the genes present in accessions 7641 and 7648 are susceptible alleles.

To validate the results obtained from *Agrobacterium tumefaciens*‐mediated transient expression assays (agroinfiltration) in *N. benthamiana*, we generated stable transgenic plants of original Rpi‐blb1/RB (Blb1‐7637) and its key variants (Blb1‐7644, 7647, and 7648) in the susceptible potato cultivar *Desiree*. PCR analysis confirmed the presence of *Rpi‐blb1/RB* and its variants in the transgenic lines (Figure S4A, B), and qRT‐PCR revealed constitutive transcript increases of the transgenes in these plants (Figure S4C). No obvious phenotypic differences were observed between WT and transgenic plants under our growth conditions (Figure S4D). Additionally, leaves showed no hypersensitive response (HR) self‐activation phenotype under UV light (Figure S4E), and qRT‐PCR analysis of the defense gene *StPR1* expression revealed no significant differences between WT and transgenic plants (Figure S4F). Subsequently, we evaluated the late blight resistance in the transgenic plants alongside non‐transgenic controls. The results demonstrated that transgenic plants expressing the original Rpi‐blb1/RB (Blb1‐7637) and functional variants (Blb1‐7644, 7647) exhibited resistance to *P. infestans*, whereas WT *Desiree* plants and those expressing the non‐functional variant (Blb1‐7648) remained susceptible (Figures 4C, D, S5). The late blight resistance observed in transgenic potato plants was consistent with the results from agroinfiltration assays in *N. benthamiana*, further underscoring the functional significance of naturally occurring NLR diversity.

### Functional analysis of *Rpi‐blb1/RB* variants in effector recognition

**Figure 5:**
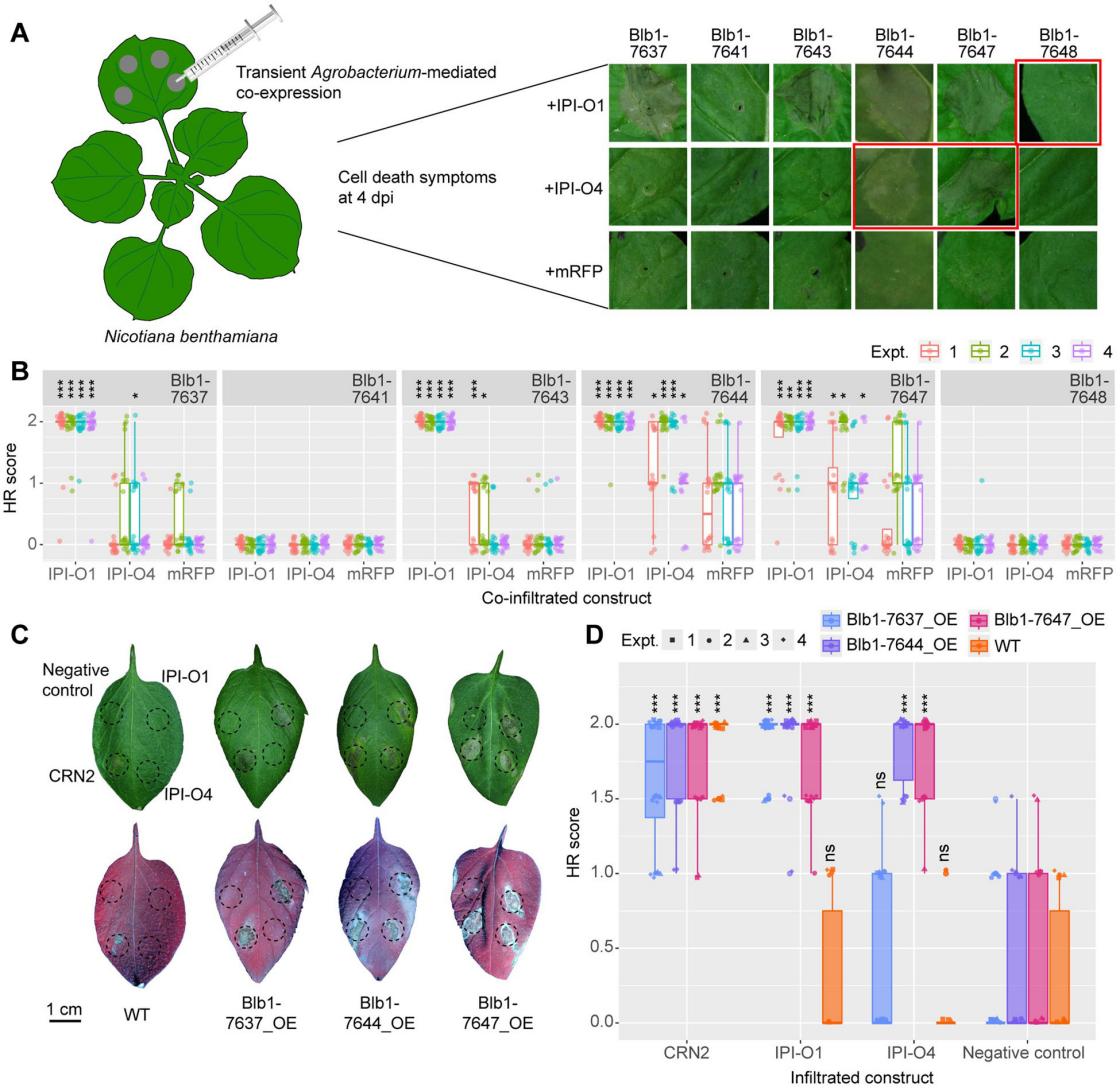
***Phytophthora infestans*** **IPI‐O1 and IPI‐O4 effector recognition by the Rpi‐blb1/RB variants** **(A)** Schematic representation of the hypersensitive response (HR) assay (left) and representative pictures of effector recognition by the Rpi‐blb1/RB variants in *N. benthamiana* (right). Rpi‐blb1/RB variants were co‐infiltrated with either *P. infestans* effectors IPI‐O1, IPI‐O4 or the control free mRFP constructs. Infiltrated leaf areas were scored for cell death 4 d post‐infiltration, 0 = no cell death, 1 = partial cell death, and 2 = complete cell death. **(B)** Statistical analysis of **(A)**. **(C)** Effector recognition in transgenic potatoes overexpressing key *Rpi‐blb1/RB* variants. HR phenotypes were recorded 4 d post‐inoculation from 0 to 2 (0: no visible symptom, 1: yellow/dirty spots, 1.5: partly necrotic, 2: fully necrotic). **(D)** Statistical analysis of **(C)**. In **(B**, **D)**, significantly different are indicated by asterisks, *P < 0.05; **P < 0.01; ***P < 0.001; means were compared with a Kruskal–Wallis rank‐sum test, if the resulting *P*‐value was < 0.05, the value was further refined with a Wilcoxon rank‐sum test with continuity correction.

To provide further support for the hypothesis that the *Rpi‐blb1/RB* variants from 7643, 7644, and 7647 encode functional resistance proteins capable of recognizing the *P. infestans* effector IPI‐O1, tag‐free versions of the NLRs and IPI‐O1 constructs were transiently co‐expressed in *N. benthamiana* leaves. In addition, the NLR constructs were also co‐infiltrated with a free mRFP construct or with the IPI‐O4 effector construct. The latter is an IPI‐O1 sequence‐related effector from *P. infestans*, which does not elicit a *Rpi‐blb1/RB*‐mediated response *in planta* (Champouret et al., 2009). Four d post‐inoculation the infiltrated leaf areas were scored for the presence of an HR on a scale of 0–2; 0 indicating no visible cell death, 1 when the infiltrated area was partly necrotic and 2 when the infiltrated area was fully necrotic. As expected, neither the Blb1‐7641 nor the Blb1‐7648 non‐functional variants triggered an HR in the presence of IPI‐O1 or IPI‐O4 (Figure 5A, B; Table S4). By contrast, the *Rpi‐blb1/RB* clones from 7637 (originally described form), 7643, 7644, and 7647 all led to a complete cell death of the infiltrated tissues, with an averaged HR score that was consistently (across four experiments) and significantly (*P* < 0.001) different from the score obtained for the NLRs expressed in the presence of the free mRFP control construct. These results demonstrate that the functional Rpi‐blb1/RB resistance proteins from the different *S. bulbocastanum* accessions were able to recognize IPI‐O1. Surprisingly, in our conditions, the originally described Rpi‐blb1/RB (Blb1‐7637) and the functional variants (Blb1‐7643, 7644, 7647) also partly responded to IP‐O4 compared to the mRFP controls. This response to IPI‐O4 was most pronounced and reproducible for the variants originating from the accessions 7644 and 7647. The development of some cell death mediated by these *Rpi‐blb1/RB* variants in the absence of the cognate effector (mRFP control) suggested that the related NLRs could be partly auto‐active in the heterologous expression system. To confirm this hypothesis, the different *S. bulbocastanum Rpi‐blb1/RB* constructs were infiltrated on their own at different concentrations of agrobacteria ( OD_600 nm_ ranging from 0.1 to 0.5) (Figure S6). The intensity of the cell‐death symptoms was dose‐dependent, confirming that the active NLRs Blb1‐7644 and Blb1‐7647 were somewhat auto‐active when infiltrated at higher OD_600 nm._ This was used to inform the experimental design in such a way that the OD_600 nm_ was adjusted to avoid autoactivation in *N. benthamiana*. In that condition, a consistent and statistically significant difference could be observed in the HR that developed in the presence of IPI‐O effectors compared to the symptoms developing in the presence of the free mRFP (Table S4).

To further elucidate the functional characteristics of *Rpi‐blb1/RB* variants, HR assays were conducted in transgenic potato lines (Blb1‐7637, 7644, 7647) alongside WT *Desiree* controls to assess their recognition capabilities. The experimental procedure involved infiltration of *Agrobacterium* (AGL1) harboring tag‐free IPI‐O1 and IPI‐O4 constructs into potato leaves. CRN2 served as a positive control, while untransformed *Agrobacterium* was utilized as a negative control. HR phenotypes were systematically evaluated 4 d post‐inoculation on a scale from 0 to 2 (0: no visible symptoms; 1: yellowing; 1.5: partial necrosis; 2: complete necrosis). The experimental results demonstrated that transgenic plants overexpressing both original Rpi‐blb1/RB (Blb1‐7637) and its variants (Blb1‐7644, 7647) exhibited pronounced cell‐death symptoms following IPI‐O1 infiltration, a response that was noticeably absent in WT *Desiree* plants (Figure 5C). Notably, when challenged with IPI‐O4, the original Rpi‐blb1/RB (Blb1‐7637) failed to induce HR, whereas both functional variants (Blb1‐7644, 7647) displayed significant HR‐inducing activity (Figure 5C, D, Table S5). Importantly, no statistically significant autoactivation was observed in the transgenic plants, which, when challenged with the negative control, responded similarly to WT *Desiree* plants. This confirms that the autoactivation observed in *N. benthamiana* was attributable to the heterologous expression system and was further exacerbated by high *Agrobacterium* infiltration densities (OD_600 nm_), highlighting the need for careful control of conditions during transient expression assays. However, together, the independent results from transient expression in *N. benthamiana* and transgenic plants confirm that active *Rpi‐blb1*/*RB* variants from *S. bulbocastanum* recognize IPI‐O1, while SNPs in variants Blb1‐7644 and Blb1‐7647 may extend this recognition to IPI‐O4.

### Combinatorial mutation analysis in Rpi‐blb1/RB identified a critical amino acid for IPI‐O1 effector recognition and epistatic interactions between variable residues

**Figure 6:**
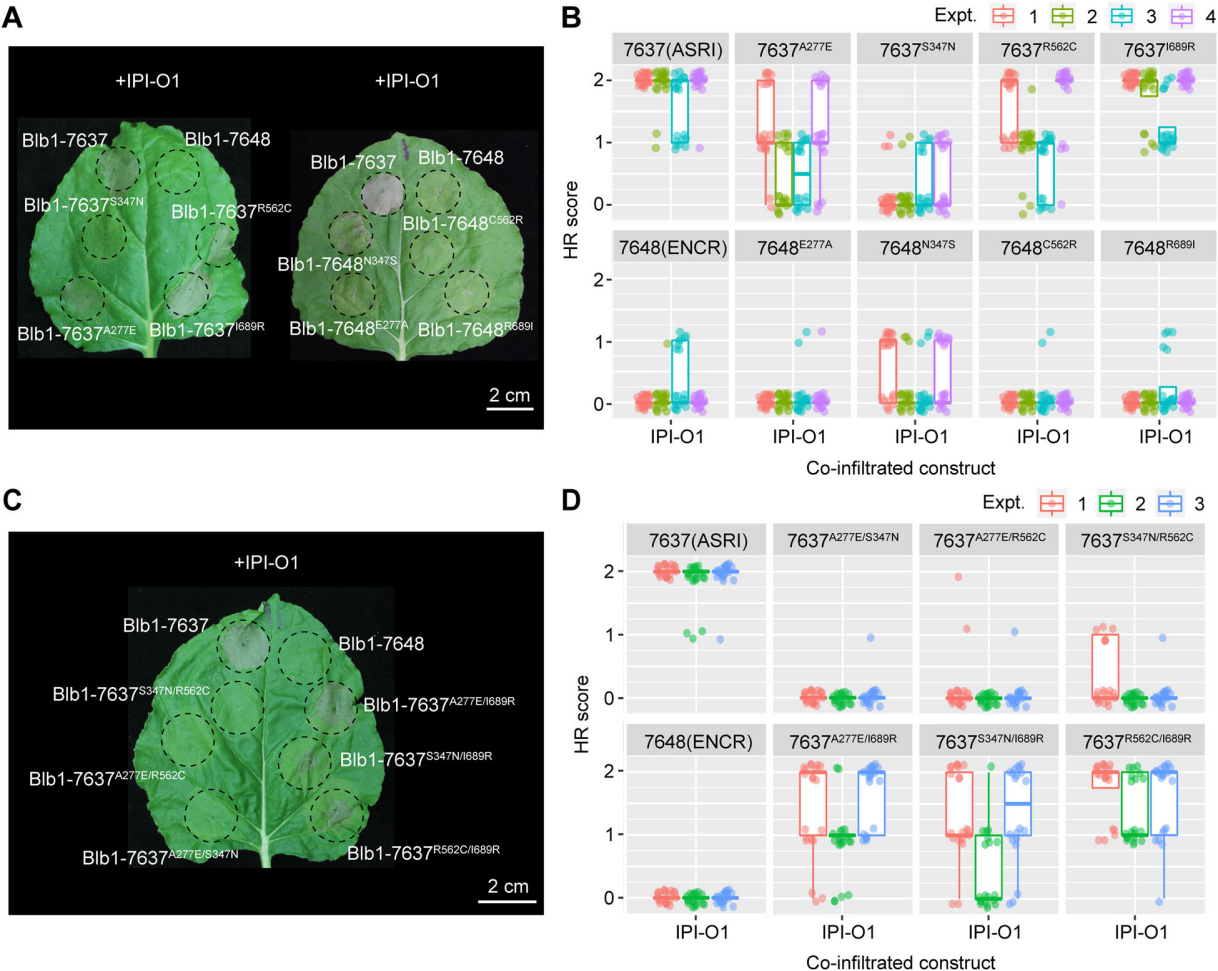
***Phytophthora infestans*** **IPI‐O1 effector recognition by the single or double amino acid mutants derived from Rpi‐blb1/RB and Blb1‐7648** **(A−D)** Cell‐death symptoms induced in *Nicotiana benthamiana* leaves by transient *Agrobacterium*‐mediated co‐expression of the Rpi‐blb1/RB single **(A)** or double **(C)** amino acid mutant constructs with the *Phytophthora infestans* IPI‐O1 effector. Statistical analyses were shown in **(B**, **D)**. HR score, 0 = no cell death, 1 = partial cell death, 2 = complete cell death in the infiltrated area.

The newly found non‐functional variant present in *S. bulbocastanum* accession 7648 contains 13 SNPs compared with the original *Rpi‐blb1/RB* gene (7637) (Table S2). These polymorphisms result in only four amino acid changes (Ala(A)277Glu(E), Ser(S)347Asn(N), Arg(R)562Cys(C), and Ile(I)689Arg(R)) that discriminate the original NLR protein (7637) from the non‐functional variant. Therefore, to determine which residues or a combination of mutations were responsible for the loss of function, a combinatorial mutation analysis was performed for which single, double and triple mutants were generated. Mutants are identified based on the nature of the four sequential variable amino acids in the sequence, as present in the resistant (ASRI) or in the susceptible (ENCR) form. The stability of each mutant protein was determined by western analysis (Figure S7). As we established that there was a strong correlation between providing resistance to *P. infestans* and recognition of the IPI‐O1 cognate effector, each *Rpi‐blb1/RB* mutant construct was tested *in planta* for effector recognition. This was achieved by transient co‐expression of the NLR mutants in *N. benthamiana* leaves with either the *IPI‐O1* or the control *mRFP* constructs (Figures 6, S8). The cell‐death symptoms were recorded on the scale of 0–2. Our experimental observations demonstrated that the Blb1‐7637^I689R^ variant maintained robust cell‐death responses to IPI‐O1, exhibiting comparable activity to Blb1‐7637, suggesting that the amino acid substitution of Ile(I) with Arg(R) at position 689 retains function (Figure 6A, B). However, most other single mutations (7637^A277E^, 7637^S347N^, 7637^R562C^) did compromise IPI‐O1 recognition and showed partial cell‐death degrees. The introduction of the susceptible amino acid at position 347 (Ser(S) to Asn(N)) into the resistant background (Blb1‐7637^S347N^) seemed to confer the most profound effect and significant difference (Figure 6A, B). Moreover, conversion of this amino acid into its resistant form in the susceptible background (Blb1‐7648^N347S^) was the only single mutation able to partially restore the recognition of IPI‐O1 (Figure 6A, B), suggesting that the serine at position 347 is the most critical amino acid for the activity of the NLR.

Further evidence of the importance of the serine at position 347 in the activity of the NLR came from analysis of the double mutants. Most double mutants including Blb1‐7637^A277E/S347N^, Blb1‐7637^S347N/R562C^, and Blb1‐7637^A277E/R562C^ completely lost function (Figure 6C, D). In addition, we found that three proteins containing I689R mutation partially retained their ability to recognize IPI‐O1 and triggered cell death. These included the Blb1‐7637^A277E/I689R^ and Blb1‐7637^R562C/I689R^ mutants that showed the strongest activity. Despite the presence of the susceptible Asn(N) at position 347, the double mutant Blb1‐7637^S347N/I689R^ also partially led to an HR (Figure 6C, D). These findings reveal that the arginine at position 689 significantly amplifies cell death in response to IPI‐O1. Interestingly, the double mutant Blb1‐7637^A277E/R562C^ (corresponding to Blb1‐7648^N347S/R689I^) harbors the resistant Ser(S) at position 347, but displayed loss of function (Figure 6C, D). This suggests that the substitution of arginine (R) at position 689 with isoleucine (I) may potentially impair NLR activity.

### S347N mutation disrupts Rpi‐blb1/RB stability and CCNB self‐association

**Figure 7:**
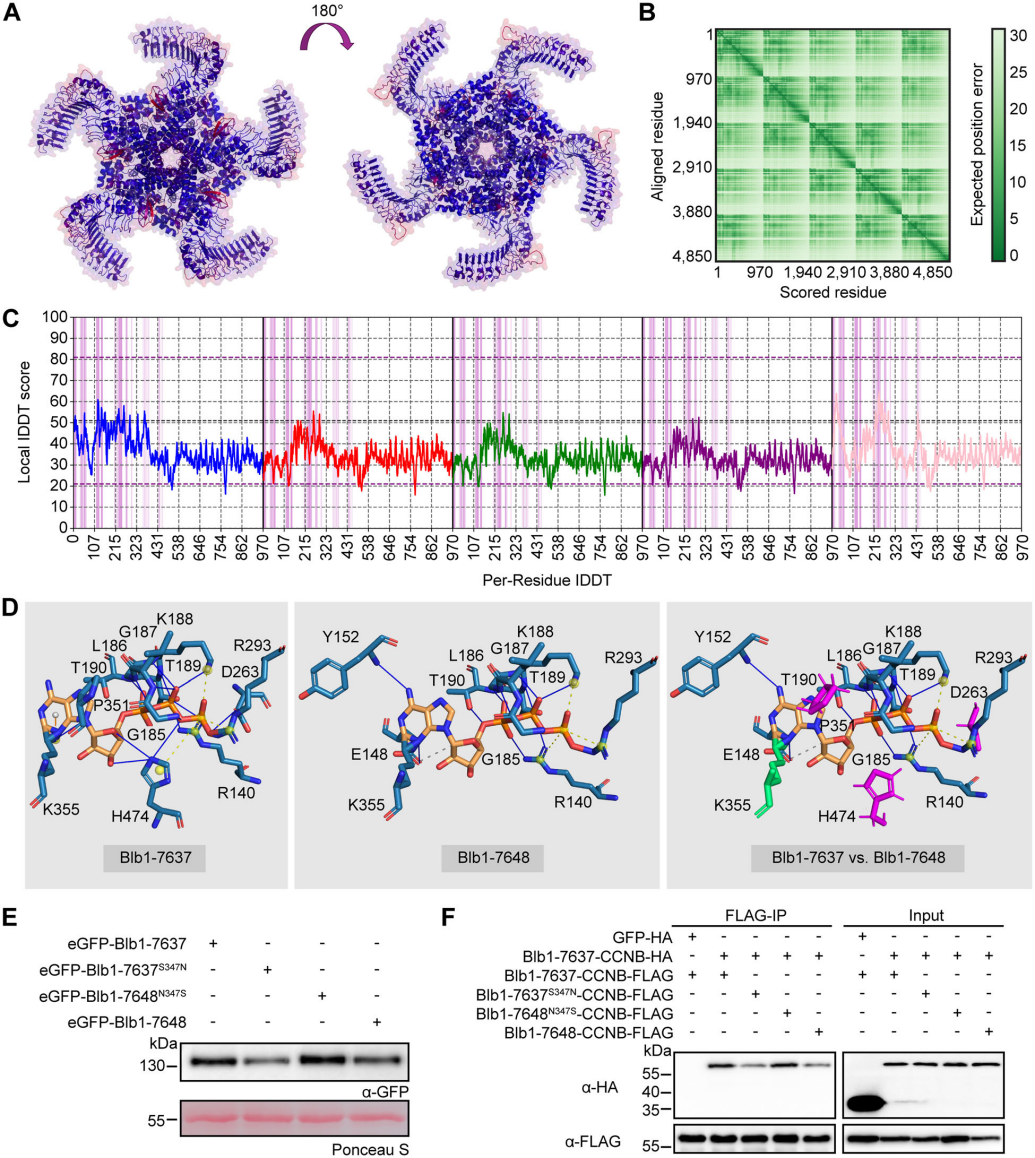
Functional analysis of the S347N mutation on pentamer formation in Rpi‐blb1/RB **(A)** The homopentameric structure of Rpi‐blb1/RB (Blb1‐7637) predicted by AlphaFold3, with an ipTM score of 0.69 and a pTM score of 0.71. The structure is colored by plDDT on a red‐blue scale (low to high confidence) and rotated 180° for visualization. **(B)** Per‐residue position error (in Ångstroms) across the predicted structure. **(C)** Residue‐level interaction analysis using DeepUMQA‐X, showing a TM‐score of 0.91, QS‐score of 0.603, Global‐lDDT of 0.347, and Interface‐lDDT of 0.37. **(D)** Rpi‐blb1/RB (Blb1‐7637) and Blb1‐7648 Protein–Ligand Interaction Comparison. Left panel shows the FunFOLD2 protein–ligand interaction prediction within the NB‐ARC domain between Blb1‐7637 and ATP, where the protein is interacting with ATP using a combination of hydrophobic, hydrogen bond and salt bridge interactions. Middle panel shows the same interaction with Blb1‐7648. Right panel shows the differences between Blb1‐7637 and Blb1‐7648 protein–ligand interaction predictions, with residues no longer predicted to be interacting shown in magenta and those with different interactions in green. **(E)** The S347N mutation destabilizes the Rpi‐blb1/RB (Blb1‐7637) protein. To assess protein stability, recombinant fusion constructs were transiently expressed in *Nicotiana benthamiana* leaves for 48 h, followed by western blot analysis of protein accumulation levels. **(F)** The S347N substitution disrupts the self‐association capacity of the CCNB domain in Rpi‐blb1/RB (Blb1‐7637). Indicated proteins were co‐expressed in *N. benthamiana* for 48 h, with protein–protein interactions subsequently analyzed through co‐immunoprecipitation (co‐IP) assays using anti‐FLAG affinity resin and immunoblotting with specific antibodies.

Structural prediction analysis of Rpi‐blb1/RB was performed using AlphaFold3. The results revealed that multiple Rpi‐blb1/RB subunits assemble into a homo‐pentamer (Figure 7A), yielding a model with an ipTM score of 0.69 and a pTM score of 0.71, indicative of high reliability. Positional accuracy was evaluated, with per‐residue errors measured in Ångstroms, further validating the structural prediction (Figure 7B). Additionally, residue‐level interactions were analyzed using DeepUMQA‐X, which confirmed strong model quality (Figure 7C). In a comparative analysis of protein–ligand interactions within the NB‐ARC domain, FunFOLD2 predictions reveal that Blb1‐7637 and Blb1‐7648 interact with ATP through hydrophobic, hydrogen bond, and salt bridge interactions, though distinct differences arise from the S347N substitution in Blb1‐7648. This substitution in Blb1‐7648 reorients P351, abolishing its hydrophobic interaction with ATP—replaced by K355, which forms a hydrophobic bond and a single hydrogen bond—while eliminating the π‐cation interaction present in the WT Blb1‐7637. Additionally, Blb1‐7648 loses critical interactions, including two hydrogen bonds from H474 to the ribose group, a salt bridge from H474 to the ATP phosphate, and a hydrogen bond from D263 to the phosphate, likely interconnected with these structural shifts (Figure 7D). Subsequent investigations demonstrated that the substitution of serine at position 347 with Asparagine in Blb1‐7637 significantly compromises the protein stability of Rpi‐blb1/RB, as supported by western blot analysis (Figures 7E, S7A). This finding is consistent with the expression levels observed in Blb1‐7648. Importantly, the N347S mutation in Blb1‐7648 restored the protein stability of Rpi‐blb1/RB, highlighting the functional significance of this residue (Figures 7E, S7A). The S347 point mutation is located within the NB‐ARC domain of Rpi‐blb1/RB. To determine whether this mutation affects self‐association, a co‐immunoprecipitation (Co‐IP) assay was conducted via agroinfiltration in *N. benthamiana*. The Co‐IP results demonstrated that the S347N mutation in Blb1‐7637 disrupts the self‐association of the CCNB domain, while the reverse mutation, N347S, in Blb1‐7648 restores the interaction level to that of the original Blb1‐7637 (Figure 7F). These findings suggest that the S347N mutation may alter the oligomerization state of Rpi‐blb1/RB, thereby preventing its pentamer‐dependent activation.

### S347N mutant abrogates IPI‐O1‐dependent plasma membrane translocation of Rpi‐blb1/RB

**Figure 8:**
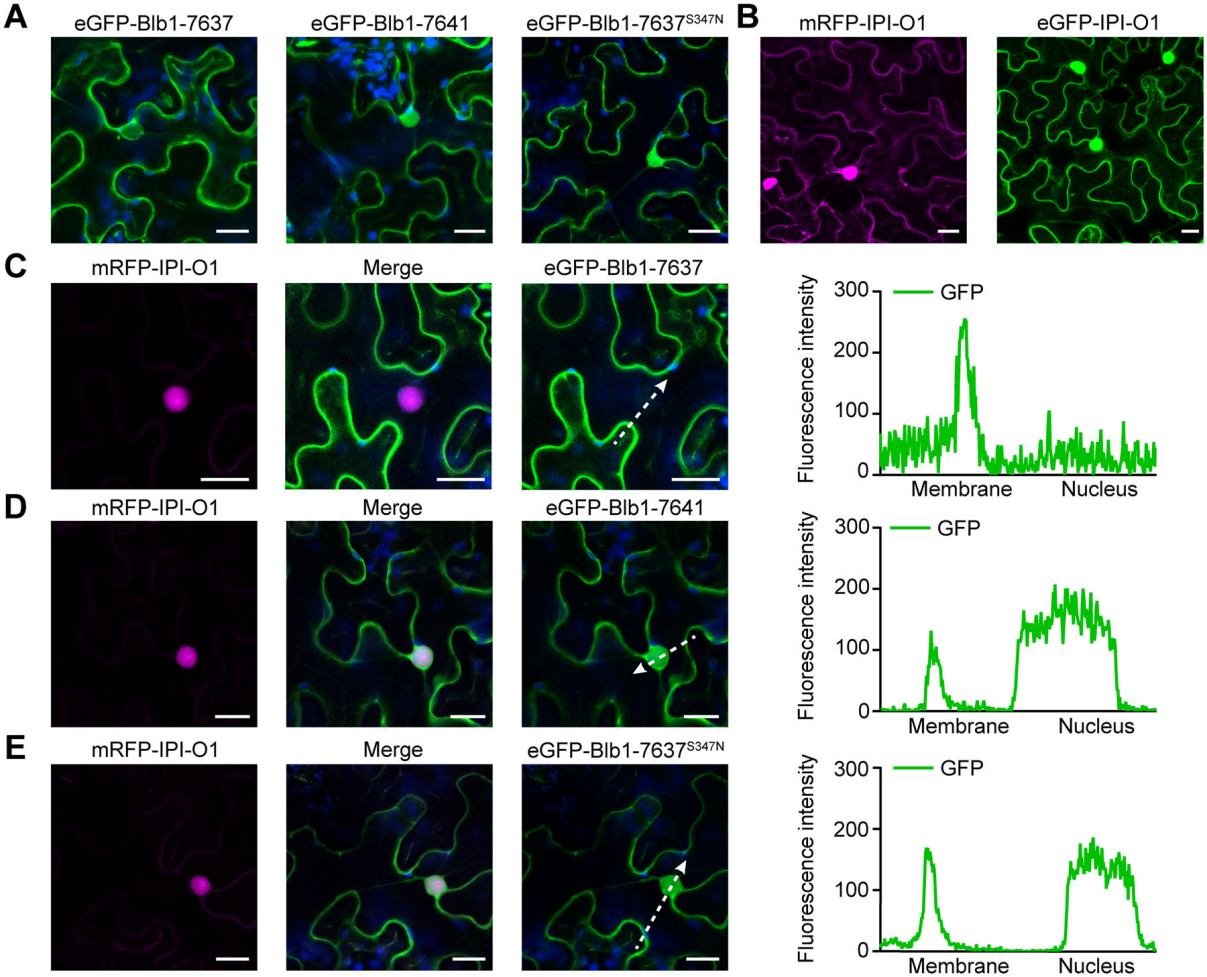
**Subcellular localization of Rpi‐blb1/RB and its non‐functional homologs in the presence or absence of** ***Phytophthora infestans*** **effector IPI‐O1** **(A**−**E)** Transient expression in *Nicotiana benthamiana* leaves of **(A)** the eGFP‐tagged original Rpi‐blb1/RB (Blb1‐7637), a non‐functional homolog (Blb1‐7641), Blb1‐7637^S347N^ mutant, and **(B)** mRFP/eGFP‐tagged effector IPI‐O1. The *P. infestans* effector protein IPI‐O1 was co‐expressed with the **(C)** Blb1‐7637, **(D)** Blb1‐7641, and **(E)** Blb1‐7637^S347N^ mutant. Indicated constructs were co‐infiltrated with mRFP or the clone carrying the p35S::mRFP::IPI‐O1 construct in pH7WGR2 in *N. benthamiana* leaves for 2–3 d. The three left panels show subcellular co‐localization patterns of different experimental combinations. Subpanel 1 shows mRFP fluorescence (magenta), subpanel 3 displays eGFP fluorescence (green) with chlorophyll autofluorescence (blue), and subpanel 2 presents merged images. The right panel illustrates the GFP fluorescence intensity profiles of Rpi‐blb1/RB and its variants across both plasma membrane and nuclear regions, with white arrows indicating the position and orientation. The scale bars represent 20 μm.

The plasma membrane localization of activated resistosome complexes following pentameric assembly is essential for triggering cell death and establishing disease resistance, as exemplified by ZAR1. To test whether Rpi‐blb1/RB exhibits analogous behavior, we investigated the subcellular localization of eGFP‐tagged WT Rpi‐blb1/RB (from *S. bulbocastanum* accession 7637) in *N. benthamiana* leaves transiently expressed with or without IPI‐O1. A truncated, non‐functional variant from accession 7641 served as a negative control. Both the original and truncated Rpi‐blb1/RB proteins fused to eGFP were cytoplasmic, while the WT Rpi‐blb1/RB (Blb1‐7637) seemed to be largely excluded from the nucleoplasm (Figure 8A). Irrespective of the presence of the NLRs, the IPI‐O1 effector fused to mRFP or eGFP mainly localized to the nucleoplasm where it strongly accumulated in the nucleolus, and partially localized in cytoplasm (Figure 8B). Our localization results for Rpi‐blb1/RB and IPI‐O1 are in line with results shown by Zhao and Song (2021). These localizations are specific to the fusion proteins as the free eGFP and mRFP proteins were both cytoplasmic and small enough to diffuse in the nucleoplasm but were excluded from the nucleolus (Figure S9). Notably, co‐expression of WT Rpi‐blb1/RB with IPI‐O1 induced NLR re‐localization to the plasma membrane (Figure 8C), a phenotype consistently observed across multiple biological replicates (*n* = 40 cells analyzed; Figure S10). This redistribution was effector‐dependent, as the truncated NLR (Blb1‐7641) remained cytoplasmic even in the presence of IPI‐O1 (Figure 8D). Functional analysis of NLR mutants revealed that the S347N mutation dramatically abolished both effector‐induced membrane targeting and nuclear exclusion, maintaining strong nuclear localization irrespective of IPI‐O1 (Figure 8A, E), while membrane re‐localizations still occurred in cells expressing IPI‐O1 and other variants (Figure S11). Given the risk of an N‐terminal GFP tag disrupting Rpi‐blb1 function, we created C‐terminal GFP‐tagged constructs for key Rpi‐blb1/RB variants. We observed consistent results in effector recognition and subcellular localization (Figure S12A–C). We confirmed the co‐localization of Blb1‐7637‐GFP with mRFP‐IPI‐O1 using AtFLS2‐BFP (PM marker) and popP2‐BFP (nuclear marker). Despite HR‐induced cell collapse, the data support our conclusions (Figure S12D). These findings demonstrate that the S347N mutation disrupts both NLR activation and membrane re‐localization, providing mechanistic insight into its loss‐of‐function phenotype and confirming the requirement for membrane association in resistance signaling.

## DISCUSSION

*Solanum bulbocastanum* is a rich source of resistance to *P. infestans* revealing known and novel resistances (Khiutti et al., 2015; Bachmann‐Pfabe et al., 2019; Duan et al., 2021; Karki et al., 2021b), which plays a vital function in the resistant breeding. In this study, the identification of 11 resistant accessions of *S. bulbocastanum* to late blight from the 12 that were screened underlines the efficacy of screening wild potato germplasm for sources of resistance (Figure 1). Of these 11 accessions, the most likely source of an entirely novel resistance is 7650 and 7641 (Figure 2). Indeed, 7650 has very low coverage of known resistances, except for *Rpi‐blb3*, that is known to be overcome by the *P. infestans* isolates of the genotype 13_A2 used in this study (Cooke et al., 2012). This resistance was recently mapped to a 2.3 Mb region on chromosome 5 (Li et al., 2023). Similarly, we concluded that 7641, which encodes only a truncated form of Rpi‐blb1/RB due to a premature stop codon, contains a novel late blight resistance and does not carry Rpi‐blb2 or any other previously characterized resistance. Therefore, this accession lends itself to genetic studies, as demonstrated for 7650.

Functional homologs of *Rpi‐blb1/RB* resistance gene family have been identified previously in the wild species *S. stoloniferum* (*Rpi‐sto1*) and *S. papita* (*Rpi‐pta1*) (Vleeshouwers et al., 2008) as well as in *S. verrucosum* (*RB* ^Ver^) (Liu and Halterman, 2006). Recently, alternative splicing of *Rpi‐blb1/RB* results in different transcriptional isoforms (*RB_IR* and *RB_CDS*) were found to regulate homeostasis between growth and immunity (Sun et al., 2024). Our findings extend this evolutionary narrative by revealing natural variation in Rpi‐blb1/RB across *S. bulbocastanum* accessions (Figure 2). There is evidence for selection at the Rpi‐blb1/RB locus as three accessions (7643, 7644, and 7647) have lost the originally described *Rpi‐blb1/RB* sequence in favor of the retention of functional variants. Furthermore, only three of the 11 resistant *S. bulbocastanum* accessions in this study (7637, 7646, and 7651) have retained the originally described Rpi‐blb1/RB sequence while two (7641 and 7648) might have lost the functional forms (Figure 2). In the accession 7641 a premature stop codon is responsible for the lack of function whereas in the accession 7648 four non‐synonymous SNPs impair Rpi‐blb1/RB. We speculate that Rpi‐blb1/RB in the latter accession could have lost function by a single mutation in a critical amino acid (e.g., Ser347) (Figure 6) and accumulated further mutations (both synonymous and non‐synonymous) as a hallmark of pseudogenization. The observed allelic diversity may represent an adaptive reservoir enabling rapid responses to shifting pathogen pressures. Systematic characterization of these variants could inform predictive models of durable disease resistance and guide precision breeding strategies.

The co‐evolution that takes place between pathogens and hosts, produces considerable evolutionary pressure on pathogens to evade recognition and on hosts to evolve new or enhanced recognition specificities. In line with a previous study (Khiutti et al., 2015), we also observed that for *S. bulbocastanum*, there is no obvious relationship between the geographic origin of accessions and their genetic variation (Figure 3). Nevertheless, our study demonstrates that all three *Rpi‐blb1/RB* variants cloned from resistant accessions 7643, 7644, and 7647 are capable of recognizing not only IPI‐O1 but partially recognizing IPI‐O4 as well, and suppressing *P. infestans* infection (4, 5). This observation aligns with the hypothesis of evolving enhanced recognition specificities, indicative of positive selection. Despite employing rigorously designed experimental protocols to assess effector‐triggered hypersensitive response (HR), there remains a possibility that the expanded recognition capabilities observed in the heterologous model plant *N. benthamiana* may not fully translate to transgenic potato plants, as previously noted in two independent studies involving *R3a*. (Chapman et al., 2014; Segretin et al., 2014). To address this potential limitation, we developed transgenic lines overexpressing two enhanced *Rpi‐blb1/RB* variants (*Blb1‐7644* and *Blb1‐7647*) alongside *Blb1‐7637* (WT *Rpi‐blb1/RB*) that demonstrated optimal HR reproducibility among the screened accessions (Figure 4). The consistent recognition responses observed in these transgenic plants underscore the novel functional properties of these variants (Figure 5). The naturally occurring functional variants discovered in this study may have utility in plant breeding as they may recognize novel variants of IPI‐O including IPI‐O4 encountered during co‐evolution with local *P. infestans* populations or provide protection by enhanced immune responses.

Structural and functional dissection of Rpi‐blb1/RB reveals pentamer‐dependent activation mechanisms. A previous study to identify variation within Rpi‐blb1/RB that affects resistance in relation to IPI‐O4, reported key variation in the CC domain (K115T and K117D) (Karki et al., 2021a), which are distinct to the five Rpi‐blb1/RB variants Blb1‐7641, Blb1‐7643, Blb1‐7644, Blb1‐7647, and Blb1‐7648. In this study, the structural modeling reveals that Rpi‐blb1/RB adopts a homologous pentameric conformation (Figures 7A–C), consistent with the predicted CC‐type NLR resistosome structure of SbRpi‐blb1 more recently reported by Madhuprakash et al. (2024). Importantly, the S347 residue is located within the NB‐ARC domain, which is essential for ATP binding and the regulation of NLR activation (Figure 7D). Experimental validation has shown that the S347N substitution in Blb1‐7637 significantly destabilizes the Rpi‐blb1/RB protein, as evidenced by western blot analyses (Figure 7E, S7A), and disrupts the self‐association of the CCNB domain (Figure 7F). These results corroborate our structural prediction of a pentamer‐dependent activation mechanism, indicating that S347 modulates Rpi‐blb1/RB oligomerization through interactions within the CCNB domain. In this study, the S347 residue was identified as a molecular switch regulating this process. Our findings enhance the structural understanding of NLR activation pathways while offering mechanistic explanations for pathogen recognition specificity in plant–pathogen interactions.

The spatiotemporal dynamics of NLR protein localization emerge as a critical regulatory layer in immune receptor activation. Here, we have observed some evidence that partial re‐localization to the plasma membrane occurs for the resistance protein upon co‐infiltration with IPI‐O1 (Figures 8C, S10B). This partial re‐localization supports the analogy with ZAR1 and is consistent with the model for ZAR1 function in which the activated pentamer is membrane associated (Wang et al., 2019). The truncated Rpi‐blb1/RB variant (Blb1‐7641) and the non‐functional S347N mutant (Blb1‐7637^S347N^) abolished IPI‐O1‐induced re‐localization, indicating that Rpi‐blb1/RB activation requires membrane‐associated processes (Figure 8D, E). Previous reports indicated a direct interaction between Rpi‐blb1/RB and the cognate effectors of the IPI‐O family, which is dependent on the CC domain (Chen et al., 2012). Thus, it is also conceivable that the discovered mutations in Rpi‐blb1/RB in the susceptible accession 7648 impair the binding between the receptor and the ligand. However, for the original Rpi‐blb1/RB gene, we see no evidence for plasma membrane localization of IPI‐O1 that might be suggestive of a more transient interaction. Interestingly, AvrAC from *Xanthomonas campestris* does not directly interact with ZAR1 or form a complex with it. Rather, AvrAC uridylates the *A. thaliana* kinase PBL2 which is recruited to a ZAR1/RKS complex prior to oligomerization and activation (Wang et al., 2019).

In line with the concept of NLR birth and death, we have identified new Rpi‐blb1/RB variants in the species *S. bulbocastanum* that display broader recognition of extant effectors as well as two variants that result in a loss of function. In conclusion, this study offers a comprehensive functional analysis of NLR diversity and illustrates how single amino acid modifications determine pathogen recognition specificity, providing an empirical basis for understanding the adaptive landscapes of plant NLR genes and for engineering durable disease resistance.

## MATERIALS AND METHODS

### Plant materials and late blight inoculation for resistance assays

*Solanum bulbocastanum* accessions from the CPC were screened for late blight resistance. Plants were assessed for resistance to late blight using detached‐leaf assays. *S. bulbocastanum* accessions 7637, 7638, 7641, 7642, 7643, 7644, 7645, 7646, 7647, 7648, 7650, and 7651 alongside the susceptible potato control *Solanum tuberosum* cv. Craig's Royal, were inoculated with *Phytophthora infestans* isolate 2009‐7654A. This isolate belongs to the clonal lineage 13_A2 (Cooke et al., 2012). In addition, plants were challenged with the isolates NL11564, NL09066, and NL12226 collected in The Netherlands. The blight isolates were propagated on fully susceptible Craig's Royal leaves and the spore inoculum was prepared as described in Andrivon et al. (2011). Disease severity was recorded on the Malcolmson scale where 1 refers to highly susceptible and 5 refers to highly resistant. The assay was repeated three times per plant and isolate.

Similarly, sets of 12 detached‐leaf replicates per resistance gene construct were inoculated with the isolate 1555c (genotype 13_A2) to assess late blight resistance conferred by *Rpi‐blb1/RB* alleles transiently expressed in *N. benthamiana* 24 h before *P. infestans* infection. Infected leaves were photographed under both white and UV light to record disease symptoms. Lesion sizes were measured on the UV‐exposed leaves. Each assay was replicated independently three times.

### RenSeq and dRenSeq analyses

Genomic DNA (1 µg) isolated from *S. bulbocastanum* accessions (7636, 7637, 7638, 7641, 7643, 7644, 7645, 7646, 7647, 7648, 7650, and 7651) was analyzed by Resistance gene enrichment and sequencing (RenSeq) using the method described by Armstrong et al. (2019). The isolated DNA was fragmented, purified and barcoded with Illumina adaptors (Next Ultra DNA library prep kit for Illumina, NEB, Ipswich, USA) before enrichment and sequencing on an Illumina MiSeq. For the diagnostic RenSeq (dRenSeq) analysis, paired‐end Illumina MiSeq reads (2×250bp) were adapter trimmed and mapped using Bowtie 2 (Langmead and Salzberg, 2012) to a reference set of known NLR genes (Armstrong et al., 2019). In diagnostic mode, Bowtie 2 settings were adjusted so only reads identical to the reference sequences mapped (‐‐score‐min L, −0.01, −0.01). To enable variants of *Rpi‐blb1/RB* to be predicted, the stringency of read mapping was relaxed (‐‐score‐min L, −0.06, −0.06), corresponding with an approximately 1% miss‐match rate. The mapped reads showing 100% coverage (at a 1% mismatch rate) of known NLR genes were studied for the presence of SNPs. This allowed sequence variants of *Rpi‐blb1/RB* to be predicted computationally. In those accessions where a novel *Rpi‐blb1/RB* sequence variant with potentially noteworthy amino acid substitutions was predicted, the novel variant was amplified by PCR, cloned, sequenced, and functionally tested.

### *k‐*mer analysis

To compare the diversity of the NLRome within *S. bulbocastanum* accessions, we employed KPop (Didelot and Ribeca, 2022), a method that enables direct comparison of unassembled sequencing read sets. The KPop analysis code is open‐source and available on GitHub: https://github.com/PaoloRibeca/KPop. Using the aforementioned RenSeq reads and a *k*‐mer length of 12, we compared the NLRomes of *S. bulbocastanum* accessions with those of *S. tuberosum* and *S. tuberosum* Group *Phureja* accessions via a “basic” KPop workflow. This involves computing *k*‐mer spectra followed by projection into Correspondence Analysis (CA) space.

Three equivalent visualizations were generated from the KPop analysis: plots showing the positions of datasets in CA space along the first two and three dimensions, and a pseudo‐phylogenetic tree derived from distances in CA space (Figure 3). Convergence analysis using *k*‐mer sizes from 9 to 12 indicated that saturation was achieved at *k* = 11.

To evaluate the impact of potential contamination, the analysis was repeated using both unfiltered and filtered reads. Filtering was performed by mapping each sequencing dataset to the RenSeq probe set using the GEM mapper (Marco‐Sola et al., 2012) with a permissive alignment protocol that accommodates sequence variability in RenSeq‐targeted regions. Specifically, each paired‐end read was split into 25 nucleotide (nt) segments, and read pairs were retained if at least three segments could be mapped to the probes, allowing up to two mismatches per segment.

On average, this filtering step removed 25% of reads per sample (range: 21%–31%; Table S1). Removing non‐probe‐mapping reads did not alter clustering patterns, but slightly tightened specific clusters, suggesting that no systematic contamination bias was present in the original RenSeq datasets.

### Plasmid constructs and site‐directed mutagenesis

The full‐length coding sequences, including introns, of the *Rpi‐blb1/RB* alleles were amplified by PCR from genomic DNA for *S. bulbocastanum* accessions 7637 (identical to the reference *Rpi‐blb1/RB* functional gene, except in the intronic region; Song et al., 2003), 7641, 7643, 7644, 7646, 7647, and 7648, using gene‐specific primers (Table S6). The *Rpi‐blb1/RB* clones were subsequently recombined into the Gateway‐compatible binary expression vectors pK7WG2 and pK7WGF2 for native and N‐terminal eGFP‐fusion constructs, respectively. The truncation of Rpi‐blb1/RB CCNB domain and its variants were cloned into expression vector pCAMBIA1300‐FLAG/HA for western blot and Co‐IP assays. Key Rpi‐blb1/RB variants (Blb1‐7637, 7641, 7637^S347N^) were cloned into a C‐terminal GFP‐fusion construct (pSuper‐GFP vector) for subcellular localization confirmation. Similarly, the *P. infestans* effector genes *IPI‐O1* and *IPI‐O4* (two variants of PITG_21388) were cloned into the indicated vectors. The cloning primers are provided in Table S6.

Single, double and triple mutants were generated using the QuikChange II XL Site‐Directed Mutagenesis Kit (Agilent Technologies, Santa Clara, USA). Triple mutants were generated by reverting 1 nt from the *S. bulbocastanum* accession 7648 into the WT form found in accession 7637, and conversely single mutants were generated by introducing mutations into the 7637 backgrounds, except for the mutation T2066G (Ile to Arg) that could only be obtained through three rounds of single nucleotide reverse mutagenesis from accession 7648. Double mutants were obtained by a second round of mutagenesis from either single or triple mutants. The mutagenic oligonucleotide primers used in the procedure are described in Table S6.

The integrity of the gene sequences for each construct as well as the reading frame for the fusions were confirmed by Sanger sequencing (primers in Table S6). For transient expression in *N. benthamiana*, the resulting binary vectors were transformed by electroporation into *Agrobacterium tumefaciens* strain GV3101 carrying the helper vector pBBR1MCS5‐VIRG‐N54D encoding the virG^N54D^ virulence factor. For the pGRAB effector clones, the agrobacteria also contained the pSOUP vector for replication as pGRAB is a derivative of pGREEN.

### Gene transient expression assay in *N. benthamiana*

*Agrobacterium*‐mediated transient expression in *N. benthamiana* was performed using YEB medium pH 7.2 (1 g/L yeast extract, 5 g/L beef extract, 5 g/L bacto‐peptone, 5 g/L sucrose and 0.5 g/L MgSO_4_.7H_2_O) instead of Luria–Bertani broth. The concentration of agrobacteria (assessed as OD_600 nm_) used for each construct as well as the infiltration combinations and designs varied depending on the experimental assay. Detailed information relevant to each experiment is provided in the figure legends.

For testing resistance to *P. infestans in planta*, the *Rpi‐blb1/RB* alleles (in pK7WG2) and the free mRFP control constructs were infiltrated at OD_600 nm_ = 0.1 on each half of the leaf and the plants were left over‐night before late blight infection. For effector recognition‐mediated cell‐death assays, the agrobacteria at OD_600 nm_ = 0.1 for the NLR construct (in pK7WG2) was co‐infiltrated with either *IPI‐O* effector construct clone (in pK7WG2 or pH7WGR2) or the free mRFP control at OD_600 nm_ = 0.1 by spotting (ca. 100 µL spots) to allow comparison of several combinations on the same leaf. Plants were infiltrated and were protected from direct sunlight until symptoms fully developed in the positive controls. For western blot analysis, the agrobacteria carrying the *NLR* gene constructs (e.g., in pK7WGF2) were individually infiltrated at OD_600 nm_ = 0.5 on full leaves in combination with a clone of the P19 silencing suppressor at OD_600 nm_ = 0.01 (final bacteria concentrations indicated for each construct); plants were agroinfiltrated and left for 48 h before sampling for protein extraction.

### Generation of overexpression transgenic plants

*Desiree*, a tetraploid potato cultivar with high susceptibility to *P. infestans*, was employed in this study as the WT and as a host for producing transgenic plants. The binary expression vector pK7WG2 constructs recombined with Blb1‐7637/7644/7647/7648 clones were transferred into *A. tumefaciens* strain GV3101. *Agrobacterium*‐mediated genetic transformation and regeneration was performed with healthy stem explants of *Desiree*, candidate transgenic plants were cultivated with proper hormones and screened by kanamycin resistance selection. Subsequently, positive transgenic plants were identified via PCR amplification and the relative gene expression levels were measured by qRT‐PCR. Primers used for selection are listed in Table S6. Wild‐type and transgenic plantlets were cultivated on Murashige and Skoog (MS) medium with 4% sucrose and 0.7% agar. Plants were then transferred to individual pots after 3 weeks.

### Late blight inoculation and cell‐death assay for transgenic plants

Leaves from 4‐ to 5‐week‐old WT and transgenic *Desiree* plants were utilized for detached‐leaf assays, as previously described. For the cell‐death assay, approximately 3‐week‐old transgenic plants were infiltrated with *Agrobacterium* strain AGL1 suspensions expressing tag‐free versions of the IPI‐O1 and IPI‐O4 constructs at an optical density (OD_600 nm_) of 0.2. CRN2 and untransformed *Agrobacterium* served as positive and negative controls, respectively. The plants were incubated at a temperature range of 23–25°C for a duration of 3–4 d. The HR results were observed and recorded on a scale from 0 to 2, where 0 indicates no visible symptoms, 1 indicates yellowing/chlorosis, 1.5 indicates partial necrosis, and 2 indicates full necrosis. Photographs were taken under both white light and UV light conditions.

### Western blotting and immunodetection

Sampling, protein extraction in denaturing conditions, and western blotting of the protein samples visualized by SDS‐PAGE on NuPAGE Novex 4%–12% Bis‐Tris gels (Life Technologies, Carlsbad, USA), as well as immunodetection, was performed.

### Co‐immunoprecipitation assay

Tissues were ground in liquid nitrogen and subsequently lysed thoroughly in an extraction buffer composed of 1 mM EDTA, 150 mM NaCl, 25 mM Tris‐HCl (pH 7.5), 10% glycerol, 2% PVPP, 0.15% NP‐40, 10 mM DTT, 1 mM PMSF, and a 1× Protease inhibitor cocktail (MCE, Shanghai, China), maintained on ice for 30 min. To obtain total protein supernatants, centrifugation was performed at 13,000 rpm for 10 min, twice. These supernatants were then incubated with Flag‐Nano‐Agarose (AlpaLifeBio, Shenzhen, China) for 2 h under gentle rotation at 4°C. Subsequently, the beads were collected by centrifuging at 2,500 rpm for 3 min and washed four times with a washing buffer containing 1 mM EDTA, 150 mM NaCl, 25 mM Tris‐HCl (pH 7.5), 10% glycerol, and 5 mM DTT. Proteins from both input and immunoprecipitation were separated using SDS‐PAGE and identified through immunoblotting with specific antibodies.

### Structural simulation

Monomeric predicted structural models for the 970aa sequences of ‘Blight resistance protein RPI’ alternatively Blb7637 (Q7XBQ9.1) and “Rpi‐blb1” alternatively Blb7648 (WKW55433.1) were created using and the IntFOLD7 server (McGuffin et al., 2023) which integrates both trRosetta2 and LocalColabFold 1.0.0 to enhance the performance of IntFOLD‐TS in predicting protein tertiary structures. Model quality of IntFOLD7‐derived structures was determined by ModFOLD9 (McGuffin et al., 2023), providing whole‐model confidence, *P*‐value, global model quality score (GMQS), predicted residue error (Å) and predicted residue accuracy (plDDT). Further quality analysis was conducted using DeepUMQA‐X (Guo et al., 2022) for by‐residue and global plDDT and stereochemical quality analysis performed with ProCheck (Laskowski et al., 2018). Ligand and ion‐binding predictions were performed by FunFOLD (Roche et al., 2013) and predicted interactions explored using the protein–ligand interaction profiler (PLIP) (Adasme et al., 2021). Multimeric models for protein–protein interaction prediction were created using AlphaFold3 (Abramson et al., 2024) and quality analysis conducted using ProCheck and DeepUMQA‐X protein complex assessment.

### Subcellular localization

Finally, for subcellular localization studies, the agrobacteria, at an OD_600 nm_ of 0.04 for the *Rpi‐blb1/RB* allele (in pK7WGF2 or pSuper‐GFP) and subcellular localization markers (PM‐marker and nuclear marker), and at an OD_600 nm_ of 0.01 for the *IPI‐O1* effector construct (in pH7WGR2), were then co‐infiltrated in the laboratory with the silencing suppressor P19 suspension (OD_600 nm_ = 0.01) and imaged 48–72 h later by confocal microscopy. Leaf segments were infiltrated with sterile distilled water, to displace air from the apoplastic spaces, before mounting on microscope slides. The abaxial leaf surface was observed using a Nikon A1R confocal laser scanning microscope mounted on a NiE upright microscope fitted with an NIR Apo 40× 0.8 W water dipping lens and GaAsP detectors. Gain settings were adjusted to prevent image saturation and ensure accurate localization. Images represent false‐colored single optical sections or maximum intensity projections of *Z*‐stacks produced using either NIS‐elements AR software or FIJI. Enhanced‐GFP (green) and chlorophyll (blue) were excited at 488 nm with emission at 500–530 and 663–737 nm, respectively, and mRFP (magenta) sequentially excited at 561 nm with emission collected at 570–620 nm. For C‐terminal GFP tagged Rpi‐blb1/RB variants, GFP (green), mRFP (magenta) and BFP (blue) were excited at 488 nm, 561 nm, 405 nm with emission at 500–530 nm, 570–620 nm, 419–476 nm, respectively. The localization experiments were repeated several times with representative images presented in the figures, compiled using ADOBE Photoshop Elements 2022.

## CONFLICTS OF INTEREST

The authors declare that they have no conflicts of interest associated with this work.

## AUTHOR CONTRIBUTIONS

I.H., X.W., and D.D. designed the research. J.L., S.M., A.K., S.G., J.Y., X.Q., K.M.W., B.H., P.R., T.C., G.M., H.L., M.F.W., T.A., S.R.F. performed the research. J.L., M.A., H.L., M.F.W., and S.M. conducted the data analysis. J.L., S.M., M.A., X.W., D.D., and I.H. wrote the manuscript. J.L. and S.M. contributed equally to this research. All authors read and approved the contents of this paper.

## Supporting information

Additional Supporting Information may be found online in the supporting information tab for this article: http://onlinelibrary.wiley.com/doi/10.1111/jipb.13950/suppinfo

**Figure S1.** Multiple sequence alignment of Rpi‐blb1/RB and the six Rpi‐blb1/RB‐like variant proteins identified in this study
**Figure S2.** Two additional replicates of the data presented in Figure 4B
**Figure S3.** Only the untagged originally described Rpi‐blb1/RB is able to trigger a hypersensitive response (HR) upon recognition of either IPI‐O1 or IPI‐O4, irrespective of the presence of a tag on the cognate effector
**Figure S4.** Identification of transgenic plants overexpressing Rpi‐blb1/RB variants
**Figure S5.** Two additional replicates of the data presented in Figure 4D
**Figure S6.** Some Rpi‐BLB1/RB allelic variants show signs of auto‐activity, leading to cell death in the absence of the cognate effector
**Figure S7.** Stability of the amino acid mutants of Rpi‐blb1/RB assessed by western blot analysis
**Figure S8.** Co‐infiltration of single or double amino acid mutants from Rpi‐blb1/RB and Blb1‐7648 with mRFP
**Figure S9.** Subcellular localization of the control free fluorescent protein tags
**Figure S10.** Subcellular localization of the resistance protein Rpi‐blb1/RB on its own or in presence of its cognate effector IPI‐O1 from *Phytophthora infestans*
**Figure S11.** Subcellular localization of the Rpi‐blb1/RB variants in the presence of the cognate effector IPI‐O1 from *Phytophthora infestans*
**Figure S12.** Rpi‐blb1/RB variants with C‐terminal GFP maintain consistent function, Rpi‐blb1/RB‐GFP and mRFP‐IPI‐O1 co‐localize with BFP‐tagged PM and nucleus markers
**Table S1.** Description of the RenSeq reads used for dRenSeq and *k*‐mer analyses
**Table S2.** Single nucleotide polymorphisms identified among the six *Rpi‐blb1/RB* variants used
**Table S3.** Details of the statistical analysis of the data presented in Figure S3
**Table S4.** Details of the statistical analysis of the data presented in Figure 5B
**Table S5.** Details of the statistical analysis of the data presented in Figure 5D
**Table S6.** Summary of primers

## Data Availability

RenSeq Illumina reads generated in this study are available at the European Nucleotide Archive (ENA) at EMBL‐EBI as project PRJEB61207. The nucleotide sequences of all *Rpi‐blb1/RB* sequences are available as GenBank accessions; Blb1‐7648 (OQ789386), Blb1‐7647 (OQ789387), Blb1‐7644 (OQ789388), Blb1‐7641 (OQ789389), Blb1‐7637 (OQ789390).
