# Supplementary material for: Solanum bulbocastanum nucleotide‐binding leucine‐rich repeat receptor evolution reveals functional variants and critical residues in Rpi‐blb1/RB
Source: J Integr Plant Biol. 2025 Jun 17;67(9):2491–509. doi: 10.1111/jipb.13950 (PMC12402751; doi:10.1111/jipb.13950)
Supplement: Supplementary file 1 — Figure S1. Multiple sequence alignment of Rpi‐blb1/RB and the six Rpi‐blb1/RB‐like variant proteins identified in this study Figure S2. Two additional replicates of the data presented in Figure 4B Figure S3. Only the untagged originally described Rpi‐blb1/RB is able to trigger a hypersensitive response (HR) upon recognition of either IPI‐O1 or IPI‐O4, irrespective of the presence of a tag on the cognate effector Figure S4. Identification of transgenic plants overexpressing Rpi‐blb1/RB variants Figure S5. Two additional replicates of the data presented in Figure 4D Figure S6. Some Rpi‐BLB1/RB allelic variants show signs of auto‐activity, leading to cell death in the absence of the cognate effector Figure S7. Stability of the amino acid mutants of Rpi‐blb1/RB assessed by western blot analysis Figure S8. Co‐infiltration of single or double amino acid mutants from Rpi‐blb1/RB and Blb1‐7648 with mRFP Figure S9. Subcellular localization of the control free fluorescent protein tags Figure S10. Subcellular localization of the resistance protein Rpi‐blb1/RB on its own or in presence of its cognate effector IPI‐O1 from Phytophthora infestans Figure S11. Subcellular localization of the Rpi‐blb1/RB variants in the presence of the cognate effector IPI‐O1 from Phytophthora infestans Figure S12. Rpi‐blb1/RB variants with C‐terminal GFP maintain consistent function, Rpi‐blb1/RB‐GFP and mRFP‐IPI‐O1 co‐localize with BFP‐tagged PM and nucleus markers Table S1. Description of the RenSeq reads used for dRenSeq and k‐mer analyses Table S2. Single nucleotide polymorphisms identified among the six Rpi‐blb1/RB variants used Table S3. Details of the statistical analysis of the data presented in Figure S3 Table S4. Details of the statistical analysis of the data presented in Figure 5B Table S5. Details of the statistical analysis of the data presented in Figure 5D Table S6. Summary of primers [file JIPB-67-2491-s001.docx]

**SUPPORTING INFORMATION**

**Figure S1. Multiple sequence alignment of *Rpi-blb1/RB* and the six *Rpi-blb1/RB*-like variant proteins identified in this study.**

**Figure S2. Two additional replicates of the data presented in Fig. 4B.**

Centre lines show the medians; box limits indicate the 25^th^ and 75^th^ percentiles as determined by R software; whiskers extend 1.5 times the interquartile range from the 25^th^ and 75^th^ percentiles; crosses represent sample means; bars indicate 95% confidence intervals of the means; data points are plotted as circles. *n* = 12 sample points. Significantly different means were calculated using two-sample *t*-tests assuming equal variances and are indicated by asterisks, *, *P* < 0.05; **, *P* < 0.01; ***, *P* < 0.001.

**Figure S3. Only the untagged originally described Rpi-blb1/RB is able to trigger a hypersensitive response (HR) upon recognition of either IPI-O1 or IPI-O4, irrespective of the presence of a tag on the cognate effector.**

**(A)** Cell death symptoms induced in *Nicotiana benthamiana* leaves by transient *Agrobacterium*-mediated co-expression of original Rpi-blb1/RB (from accession 7637) and cognate *Phytophthora infestans* effectors IPI-O1 or IPI-O4, recorded 5 days post inoculation (inoculum infiltrated at OD_600nm_ 0.1 for each construct). Symptoms observed under white light, UV light and after trypan blue staining, from left to right panels. Pictures under UV light were taken from abaxial side of the leaves and images were mirrored to observe spots with the same location as on white light images. The HR leading to cell death upon recognition of the effector was assessed using either native or tagged proteins; N-terminal fusion with the enhanced green fluorescent protein (eGFP) for Rpi-blb1/RB or with the monomeric red fluorescent protein (mRFP) for IPI-O effectors. The free mRFP was used as control to allow for detection of Rpi-blb1/RB auto-activity that may lead to cell-death in absence of effector. A set of controls, where the IPI-O effectors were expressed on their own in presence of free eGFP, is also presented. **(B)** The HR score is based on a scale of 0 (no symptoms) to 2 (fully established HR), depending on the severity and expansion of the observed symptoms under white light. Each construct combination was infiltrated 20 times across 10 plants. Two replicates are presented. Significantly different means relative to mRFP are indicated by asterisks, ***, *P* < 0.001. Detailed statistics are provided in Table S4.

**Figure S4. Identification of transgenic plants overexpressing Rpi-blb1/RB variants.**

**(A)** The PCR identification of *Kanamycin resistance* gene from pK7WG2 vector. Genomic DNA were extracted and used for PCR assay. N: Negative control, ddH_2_O. 1: wild type. 2: Blb1-7637_OE #1. 3: Blb1-7637_OE #2. 4: Blb1-7644_OE #1. 5: Blb1-7644_OE #2. 6: Blb1-7647_OE #1. 7: Blb1-7647_OE #2. 8: Blb1-7648_OE #1. 9: Blb1-7648_OE #2. P: Positive control, pK7WG2 vector. **(B)** The PCR identification of *Rpi-blb1/RB* gene. N: Negative control, ddH_2_O. 1: wild type. 2: Blb1-7637_OE #1. 3: Blb1-7637_OE #2. 4: Blb1-7644_OE #1. 5: Blb1-7644_OE #2. 6: Blb1-7647_OE #1. 7: Blb1-7647_OE #2. 8: Blb1-7648_OE #1. 9: Blb1-7648_OE #2. P1-4: Positive control, pK7WG2-Blb1-7637/7644/7647/7648 vector. **(C)** Relative transcript level of *Rpi-blb1/RB* variants in stable transgenic potatoes. **(D)** Growth phenotype of stable transgenic potatoes containing different *Rpi-BLB1/RB* variants. **(E)** Uninfected transgenic plant leaves observed under UV light. **(F)** Relative expression of *StPR1* in stable transgenic plants by qRT-PCR. Data represent mean ± SEM, *n* = 3 biologically independent samples. *P* < 0.05, One-way ANOVA followed by Tukey’s post hoc test.

**Figure S5. Two additional replicates of the data presented in Fig. 4D.**

Centre lines show the medians; box limits indicate the 25^th^ and 75^th^ percentiles as determined by R software; whiskers extend 1.5 times the interquartile range from the 25^th^ and 75^th^ percentiles; crosses represent sample means; bars indicate 95% confidence intervals of the means; data points are plotted as circles. Upper panel: *n* = 30, 12, 11, 12, 10, 8, 12, 12, 5 sample points. Lower panel: *n* = 8, 6, 6, 4, 4, 6, 6, 6, 6 sample points. Significantly different means were calculated using two-sample *t*-tests assuming equal variances and are indicated by asterisks, ***, *P* < 0.001.

**Figure S6. Some** **Rpi-BLB1/RB allelic variants show signs of auto-activity, leading to cell death in the absence of the cognate effector.**

Cell death symptoms induced in *Nicotiana benthamiana* leaves by transient *Agrobacterium*-mediated expression of six Rpi-blb1/RB allelic variants were recorded 4 days post inoculation. Infiltration with a serial dilution of inoculum from OD_600nm_ = 0.1 to 0.5 for each Rpi-blb1/RB clone or infiltration buffer including bacteria without construct as control; design of spot infiltration represented on the top left image. Symptoms observed under white light (top panel) and UV light (bottom panel). Pictures under UV light were taken from abaxial side of the leaves and images were mirrored to observe spots with the same location as on white light images. The symptoms were assessed for native proteins. Each construct was infiltrated 18-20 times across 9-10 plants. Images are representative of each Rpi-blb1/RB set. Scale bars, 1 cm.

**Figure S7. Stability of the amino acid mutants of Rpi-blb1/RB assessed by Western blot analysis.**

Stability of the single **(A)** or double **(B)** amino acid mutants derived from Rpi-blb1/RB (Blb1-7637) and Blb1-7648 assessed by Western-blot analysis. Top panel: eGFP tag detected using GFP antibodies (α-GFP). Bottom panel: total amount of proteins loaded per sample presented after transfer by staining with Ponceau red.

**Figure S8. Co-infiltration of single or double amino acid mutants from Rpi-blb1/RB and Blb1-7648 with mRFP.**

Cell death symptoms induced in *Nicotiana benthamiana* leaves by transient *Agrobacterium*-mediated co-expression of the Rpi-blb1/RB single **(A)** or double **(C)** amino acid mutant constructs with the control free mRFP constructs. Statistical analyses were shown in **(B)** and **(D)**. HR score, 0 = no cell death, 1 = partial cell death, 2 = complete cell death in the infiltrated area.

**Figure S9. Subcellular localisation of the control free fluorescent protein tags. Transient expression of agrobacteria clones, carrying either the eGFP or the mRFP constructs in pK7WG2, in *Nicotiana benthamiana* leaves either alone (A-F) or in combination (G-I).**

*Agrobacteria* infiltrated at OD_600nm_ 0.01, final concentration for each construct. The enhanced green fluorescent protein (eGFP) and the monomeric red fluorescent protein (mRFP) are cytoplasmic and can both diffuse passively to the nucleoplasm where they are excluded from the nucleolus. **(A-C)** eGFP; **(D-F)** mRFP; **(G-I)** eGFP and mRFP co-expressed; eGFP (green) in subpanels 1, mRFP (magenta) in subpanels 2 and the overlay in subpanels 3, along with chlorophyll fluorescence (blue). Nuclei images **(C, F, I)** are single optical sections selected from Z-stacks showing nucleoli (scale bars represent 2.5 µm), the other images are maximum intensity projections for whole cell images (**A, D, G**; scale bars represent 20 µm) and images focussing on the cytoplasm (**B, E, H**; scale bars represent 5 µm).

**Figure S10. Subcellular localisation of the resistance protein Rpi-blb1/RB on its own or in presence of its cognate effector IPI-O1 from *Phytophthora infestans*.**

Transient expression in *Nicotiana benthamiana* leaves of the originally described resistance protein Rpi- blb1/RB from *Solanum bulbocastanum* (accession 7637) either alone **(Panel A)** or in combination with the *P. infestans* effector protein IPI-O1 **(Panel B)**. The agrobacteria clone carrying the p35S::eGFP::*Rpi-BLB1/RB* construct in pK7WGF2 was infiltrated alone or co-infiltrated with the clone carrying the p35S::*mRFP::IPI-O1* construct in pH7WGR2 and imaged by confocal microscopy 2 and 3 days post inoculation (images 1-20 and 21-40, respectively in each panel). In both panels, images of the enhanced green fluorescent protein (eGFP; green) signal corresponding to the eGFP-Rpi-blb1/RB fusion protein, along with chlorophyll fluorescence (blue) are displayed as maximum intensity projections. The scale is the same for all images on each panel and therefore the scale bar is only indicated on the last picture and represents 10 µm.

Figure S11. Subcellular localisation of the Rpi-blb1/RB varieties in presence of the cognate effector IPI-O1 from Phytophthora infestans.

**(A)** Transient expression in *Nicotiana benthamiana* leaves of the eGFP tagged, Blb1-7637^A277E^, Blb1-7637^R562^, Blb1-7637^I689R^ mutant. The *P. infestans* effector protein IPI-O1 were co-expressed with the **(B)** Blb1-7637^A277E^, **(C)** Blb1-7637^R562^, **(D)** Blb1-7637^I689R^ mutant. The scale bars represent 20 µm.

Figure S12. Rpi-blb1/RB variants with C-terminal GFP maintain consistent function, Rpi-blb1/RB-GFP and mRFP-IPI-O1 co-localize with BFP-tagged PM and nucleus markers.

**(A)** Effector recognition by Rpi-blb1/RB variants tagged with C-terminal GFP in *N. benthamiana*. (A-1) Rpi-blb1/RB (Blb1-7637)-GFP was expressed alone or co-expressed with free mRFP, mRFP-IPI-O1, or IPI-O1. (A-2) mRFP-IPI-O1 was co-expressed with Blb1-7637-GFP or Blb1-7641-GFP. (A-3) mRFP-IPI-O1 was co-expressed with Blb1-7637-GFP, Blb1-7637^S347N^-GFP, Blb1-7648^N347S^-GFP, or Blb1-7648-GFP. **(B-C)** Subcellular localization of Rpi-blb1/RB variants with C-terminal GFP, with or without IPI-O1. **(B)** Subcellular localization of Blb1-7637, Blb1-7641, and Blb1-7637^S347N^ with C-terminal GFP. **(C)** IPI-O1 was co-expressed with Blb1-7637, Blb1-7641, and Blb1-7637^S347N^ tagged with C-terminal GFP. Scale bars, 20 µm. **(D)** Blb1-7637-GFP and mRFP-IPI-O1 co-localize with BFP-tagged PM (AtFLS2) and nucleus (popP2) markers. Subpanel 1 shows BFP, subpanel 2 shows GFP (green), and subpanel 3 shows mRFP (magenta). The right panel illustrates fluorescence intensity profiles, with white arrows indicating position and orientation. Scale bars, 20 µm.

**Table S1. Description of the RenSeq reads used for dRenSeq and k-*mer* analyses.**

| **Accession** | **Original reads** | **Reads mapping to baits** | **Surviving reads (%)** | **Description** |
| --- | --- | --- | --- | --- |
| H2-P55-7 | 6908084 | 5377224 | 77 | *S. tuberosum* |
| H2-Picasso | 9267152 | 6868508 | 74 | *S. tuberosum* |
| H2-Resistant-bulk | 8957748 | 6443184 | 71 | *S. tuberosum* |
| H2-Susceptible-bulk | 8881076 | 6804584 | 76 | *S. tuberosum* |
| DB337(73) | 15125536 | 11140556 | 73 | *S. tuberosum Group Phureja* |
| 99FT.1b5 | 4914768 | 3786548 | 77 | *S. tuberosum Group Phureja* |
| HB171(13) | 4493368 | 3432116 | 76 | *S. tuberosum Group Phureja* |
| Blb7637 | 3891736 | 2892232 | 74 | *S. bulbocastanum* |
| Blb7638 | 4448792 | 3213280 | 72 | *S. bulbocastanum* |
| Blb7641 | 3998472 | 3057804 | 76 | *S. bulbocastanum* |
| Blb7642 | 4095996 | 3124604 | 76 | *S. bulbocastanum* |
| Blb7643 | 4070724 | 3086636 | 75 | *S. bulbocastanum* |
| Blb7644 | 4939236 | 3419096 | 69 | *S. bulbocastanum* |
| Blb7645 | 6610016 | 4756640 | 71 | *S. bulbocastanum* |
| Blb7646 | 4395432 | 3278952 | 74 | *S. bulbocastanum* |
| Blb7647 | 2859036 | 2276556 | 79 | *S. bulbocastanum* |
| Blb7648 | 3520356 | 2699792 | 76 | *S. bulbocastanum* |
| Blb7650 | 3408460 | 2622200 | 76 | *S. bulbocastanum* |
| Blb7651 | 4825620 | 3602684 | 74 | *S. bulbocastanum* |

**Table S2. Single nucleotide polymorphisms identified amongst the six *Rpi-blb1/RB* variants used.** Codons in red indicate non-synonymous amino acid substitutions.

| **Position** | **ref** | **alt** | **ref codon** | **alt codon** | **7637** | **7641** | **7643** | **7644** | **7647** | **7648** | **RB** |
| --- | --- | --- | --- | --- | --- | --- | --- | --- | --- | --- | --- |
| 24 | C | T | CTG(L) | TTG(L) |  |  | 7643 |  |  |  |  |
| 27 | C | T | CTA(L) | TTA(L) |  | 7641 | 7643 | 7644 | 7647 | 7648 |  |
| 41 | T | G | ACT(T) | ACG(T) |  |  |  |  |  | 7648 |  |
| 64 | T | C | GTA(V) | GCA(A) |  | 7641 | 7643 | 7644 | 7647 |  |  |
| 122 | T | C | ATT(I) | ATC(I) |  |  |  |  |  | 7648 |  |
| 128 | C | G | GCC(A) | GCG(A) |  |  | 7643 |  |  |  |  |
| 454 | A | T | INTRON |  |  |  | 7643 |  |  |  |  |
| 487 | C | G | INTRON |  | 7637 |  |  |  |  |  | Rpi_RB |
| 500 | G | ΔG | INTRON |  |  |  |  |  |  | 7648 |  |
| 524 | C | T | INTRON |  |  | 7641 |  |  |  |  |  |
| 537 | T | A | INTRON |  | 7637 | 7641 | 7643 | 7644 | 7647 | 7648 | Rpi_RB |
| 548 | C | T | INTRON |  |  |  |  | 7644 |  |  |  |
| 549 | G | A | INTRON |  |  |  |  |  | 7647 |  |  |
| 602 | T | C | INTRON |  |  |  | 7643 |  |  |  |  |
| 707 | T | G | INTRON |  | 7637 |  |  |  |  |  | Rpi_RB |
| 814 | A | G | INTRON |  |  |  |  |  |  | 7648 |  |
| 860 | C | T | INTRON |  |  |  |  |  | 7647 |  |  |
| 885 | - | A(ins) | INTRON |  |  |  |  |  |  | 7648 |  |
| 904 | T | G | INTRON |  |  |  |  |  | 7647 |  |  |
| 1000 | T | ΔT | INTRON |  |  |  |  | 7644 |  |  |  |
| 1508 | C | A | GCA(A) | GAA(E) |  |  |  |  |  | 7648 |  |
| 1538 | C | A | TCT(S) | TAT(Y) |  |  | 7643 |  |  |  |  |
| 1718 | G | A | AGT(S) | AAT(N) |  |  |  |  |  | 7648 |  |
| 1792 | C | T | CAT(H) | TAT(Y) |  |  |  | 7644 |  |  |  |
| 2040 | C | G | TAC(Y) | TAG(*) |  | 7641641 |  |  |  |  |  |
| 2118 | G | A | TTG(L) | TTA(L) |  |  |  |  |  | 7648 |  |
| 2289 | A | G | ACA(T) | ACG(T) |  |  | 7643 |  |  |  |  |
| 2362 | C | T | CGT(R) | TGT(C) |  |  |  |  |  | 7648 |  |
| 2744 | T | G | ATA(I) | AGA(R) |  |  |  |  |  | 7648 |  |
| 2808 | A | G | TTA(L) | TTG(L) |  |  | 7643 |  |  |  |  |
| 2980 | G | C | GAG-E | CAG(Q) |  |  | 7643 |  |  |  |  |
| 3020 | T | C | TTC(F) | TCC(S) |  |  |  |  | 7647 |  |  |
| 3133 | A | C | ATG(M) | CTG(L) |  |  | 7643 |  | 7647 |  |  |
| 3179 | G | C | AGG(R) | ACG(T) |  |  | 7643 |  |  |  |  |
| 3313 | T | C | TTG(L) | CTG(L) |  | 7641 |  |  |  | 7648 |  |
| 3375 | C | T | CTC(L) | CTT(L) |  |  |  |  | 7647 |  |  |
| 3441 | A | G | CTA(L) | CTG(L) |  | 7641 |  |  |  |  |  |
| 3570 | T | G | CCT(P) | CCG(P) |  |  |  | 7644 |  |  |  |
| 3571 | A | T | AAT(N) | TAT(Y) |  |  |  | 7644 |  |  |  |
| 3574 | G | C | GTG(V) | CTG(L) |  |  |  | 7644 |  |  |  |
| 3577 | A | C | AAT(N) | CAT(H) |  |  |  | 7644 |  |  |  |
| 3582 | A | T | ATA(I) | ATT(I) |  |  |  | 7644 |  |  |  |
| 3583 | T | C |  |  |  |  |  |  |  |  |  |
| 3584 | A | G | TAT(Y) | CGT(R) |  |  |  | 7644 |  |  |  |
| 3587 | T | A | ATT(I) | AAT(N) |  |  |  |  | 7647 |  |  |
| 3588 | T | C | ATT(I) | AAC(N) |  |  |  | 7644 |  |  |  |
| 3588 | - | TAT(ins) | - | TAT(Y) |  |  |  | 7644 |  |  |  |

**Table S3. Details of the statistical analysis of the data presented in Figure S3.** Means were compared with a Kruskal-Wallis rank sum test. If the resulting *P*-value was < 0.05, the value was further refined with a Wilcoxon rank sum test with continuity correction. The data from both replicates were combined before analysis.

| **BLB1 Construct** | **Treatment** | | | | | |
| --- | --- | --- | --- | --- | --- | --- |
|  | **IPI-O1** | | **mRFP-IPI-O1** | | **mRFP** | |
| Blb1-7637 | mean | 1.825 |  | 2.0 |  | 0.25 |
|  | median | 2.0 |  | 2.0 |  | 0.0 |
|  | *P*-value IPI-O1 vs. mRFP | 1.1e-14 | *P*-value IPI-O1 vs. mRFP-IPI-O1 | 0.012 | *P*-value mRFP-IPI-O1 vs. mRFP | 2.0e-16 |
| eGFP-Blb1-7637 | mean | 0.0 |  | 0.0 |  | 0.0 |
|  | median | 0.0 |  | 0.0 |  | 0.0 |
|  | *P*-value IPI-O1 vs. mRFP | NA | *P*-value IPI-O1 vs. mRFP-IPI-O1 | NA | *P*-value mRFP-IPI-O1 vs. mRFP | NA |

| **BLB1 Construct** | **Treatment** | | | | | |
| --- | --- | --- | --- | --- | --- | --- |
|  | **IPI-O4** | | **mRFP-IPI-O4** | | **RFP** | |
| Blb1-7637 | mean | 0.76 |  | 1.42 |  | 0.026 |
|  | median | 1.0 |  | 2.0 |  | 0.0 |
|  | *P*-value IPI-O4 vs. mRFP | 4.7e-8 | *P*-value IPI-O4 vs. mRFP-IPI-O4 | 0.00026 | *P*-value mRFP-IPI-O4 vs. mRFP | 2.2e-11 |
| eGFP-Blb1-7637 | mean | 0.0 |  | 0.0 |  | 0.0 |
|  | median | 0.0 |  | 0.0 |  | 0.0 |
|  | *P*-value IPI-O4 vs. mRFP | NA | *P*-value IPI-O4 vs. mRFP-IPI-O1 | NA | *P*-value mRFP-IPI-O4 vs. mRFP | NA |

| **BLB1 Construct** | **Treatment** | **IPI-O1**  **(*P-value*: IPI-O1 vs. IPI-O4)** | | | | **IPI-O4**  **(*P-value*: IPI-O4 vs. mRFP)** | | | | **mRFP**  **(*P-value*: IPI-O1 vs. mRFP)** | | | |
| --- | --- | --- | --- | --- | --- | --- | --- | --- | --- | --- | --- | --- | --- |
|  |  | **Exp. 1** | **Exp. 2** | **Exp. 3** | **Exp. 4** | **Exp. 1** | **Exp. 2** | **Exp. 3** | **Exp. 4** | **Exp. 1** | **Exp. 2** | **Exp. 3** | **Exp. 4** |
| Blb1-7637 | mean | 1.85 | 1.9 | 1.95 | 1.9 | 0.15 | 0.65 | 0.45 | 0.1 | 0.05 | 0.45 | 0.1 | 0 |
|  | median | 2 | 2 | 2 | 2 | 0 | 0 | 0 | 0 | 0 | 0 | 0 | 0 |
|  | *P-value* | 3.60E-08 | 8.90E-08 | 6.60E-08 | 1.30E-08 | 0.31 | 0.59 | 0.03 | 0.16 | 2.60E-08 | 9.50E-08 | 6.00E-09 | 9.40E-08 |
| Blb1-7641 | mean | 0 | 0 | 0 | 0 | 0 | 0 | 0 | 0 | 0 | 0 | 0 | 0 |
|  | median | 0 | 0 | 0 | 0 | 0 | 0 | 0 | 0 | 0 | 0 | 0 | 0 |
|  | *P-value* | NA | NA | NA | NA | NA | NA | NA | NA | NA | NA | NA | NA |
| Blb1-7643 | mean | 2 | 2 | 2 | 2 | 0.55 | 0.4 | 0.15 | 0 | 0.05 | 0.1 | 0.1 | 0.05 |
|  | median | 2 | 2 | 2 | 2 | 1 | 0 | 0 | 0 | 0 | 0 | 0 | 0 |
|  | *P-value* | 6.30E-09 | 5.90E-09 | 2.30E-09 | 1.10E-09 | 7.00E-04 | 0.032 | 0.65 | 0.34 | 2.20E-09 | 3.30E-09 | 2.30E-09 | 1.10E-09 |
| Blb1-7644 | mean | 2 | 1.95 | 2 | 2 | 1.2 | 1.95 | 1.85 | 1 | 0.7 | 1.1 | 0.75 | 0.6 |
|  | median | 2 | 2 | 2 | 2 | 1 | 2 | 2 | 1 | 0.5 | 1 | 1 | 1 |
|  | *P-value* | 3.10E-05 | 1 | 0.08 | 2.50E-07 | 0.042 | 1.60E-06 | 3.20E-05 | 0.036 | 2.50E-06 | 1.60E-06 | 2.50E-06 | 5.40E-08 |
| Blb1-7647 | mean | 1.7 | 1.95 | 1.9 | 2 | 0.9 | 1.85 | 0.95 | 1 | 0.35 | 1.4 | 0.6 | 0.6 |
|  | median | 2 | 2 | 2 | 2 | 1 | 2 | 1 | 1 | 0 | 1 | 0 | 1 |
|  | *P-value* | 0.0019 | 0.3 | 1.80E-06 | 2.50E-07 | 0.0174 | 0.0126 | 0.11 | 0.036 | 1.10E-05 | 0.0021 | 5.40E-06 | 5.40E-08 |
| Blb1-7648 | mean | 0 | 0 | 0.05 | 0 | 0 | 0 | 0 | 0 | 0 | 0 | 0 | 0 |
|  | median | 0 | 0 | 0 | 0 | 0 | 0 | 0 | 0 | 0 | 0 | 0 | 0 |
|  | *P-value* | NA | NA | NA | NA | NA | NA | NA | NA | NA | NA | NA | NA |

**Table S4. Details of the statistical analysis of the data presented in Figure 5B. Means were compared with a Kruskal-Wallis rank sum test.** If the resulting *P-value* was < 0.05, the value was further refined with a Wilcoxon rank sum test with continuity correction.

**Table S5. Details of the statistical analysis of the data presented in Figure 5D. Means were compared with a Kruskal-Wallis rank sum test.** If the resulting *P-value* was < 0.05, the value was further refined with a Wilcoxon rank sum test with continuity correction.

| **Co-infiltrated construct** | **Statistic** | **7637_OE** | **7644_OE** | **7647_OE** | **WT** |
| --- | --- | --- | --- | --- | --- |
| CRN2 | mean | 1.63 | 1.75 | 1.79 | 1.89 |
|  | median | 1.75 | 2.00 | 2.00 | 2.00 |
|  | Adjusted *P*-value | 6.80E-08 | 1.70E-06 | 7.60E-07 | 3.80E-06 |
| IPI-O1 | mean | 1.94 | 1.92 | 1.76 | 0.29 |
|  | median | 2.00 | 2.00 | 2.00 | 0.00 |
|  | Adjusted *P*-value | 4.20E-09 | 5.40E-07 | 7.60E-07 | 1.00 |
| IPI-O4 | mean | 0.42 | 1.86 | 1.82 | 0.14 |
|  | median | 0.00 | 2.00 | 2.00 | 0.00 |
|  | Adjusted *P*-value | 0.32 | 5.90E-07 | 5.90E-07 | 0.47 |
| Negative control | mean | 0.23 | 0.42 | 0.37 | 0.29 |
|  | median | 0.00 | 0.00 | 0.00 | 0.00 |
|  | Adjusted *P*-value | NA | NA | NA | NA |

**Table S6. Sequences of primers used for cloning the *Rpi-blb1/RB* gene variants, the two *IPI-O* effectors (variants of PITG_21388) and the *mRFP* control gene, as well as for sequencing all the constructs.** Start and Stop codons included in cloning primers, as well as single nucleotide polymorphism (SNP) found in primers used for site-directed mutagenesis are indicated in bold and underlined.

| **Primer name** | **Sequence 5’->3’** | **Primer use** |
| --- | --- | --- |
| RFP-ATG-FOR | ACC**ATG**GCCTCCTCCGAGG | Cloning the *mRFP* control gene |
| RFP-TAA-REV | **TTA**GGCGCCGGTGGA |  |
| 21388-SP_F | AAAGCAGGCTTCACC**ATG**GTTTCATCCAATCTC | Cloning PITG_21388 variants  IPI-O1/4 without signal peptide |
| IPI01R | GAAAGCTGGGTC**CTA**GCTAGGGCCAACGTT |  |
| Blb1_var-F | CCAACA**ATG**GCTGAAGCTTTCATTCAAGTTCTG | Cloning *Rpi**-blb1/RB* variants |
| Blb1_var-R | CTCACAAACAAAGAAACAATAGCAAATAAC**TTA**AA |  |
| Blb1_var1-R | CTCACAAACAAAGAAACAATAGCAAATAAC**TTA**AT |  |
| Blb1_var2-R | ATA**TTA**ATAGTTACGAATATGCAGATACGGAATGT |  |
| CCNB-F | AGCAGGCTTCGAATTCATGGCTGAAGCTTTCATTCA | Cloning CCNB domain of *Rpi-blb1/RB* variants |
| CCNB-R | AAGCTGGGTTCTCGAGGGGGGGAAGAGTGTAAAAAAAC |  |
| Blb1seq-F1 | TATCCCTTTCCGTCACAAGG | Sequencing *Rpi-blb1/RB* constructs Internal primers |
| Blb1seq-F2 | CACTCGATCCGTTTTGCTTTTC |  |
| Blb1seq-F3 | GCCCAACACCTTTCAGTCCT |  |
| Blb1seq-F4 | GAAGATTGTTGGTTGTTGTTC |  |
| Blb1seq-F5 | AAGATGGTAAAACTTATTTCAAGATG |  |
| Blb1seq-F6 | AGGATCATTGACATGCCTTA |  |
| Blb1seq-F7 | GAAGTGGATATTGATGTTCATTC |  |
| Blb1seq-F8 | AAATGTTTACCAGAGGGATTGC |  |
| Blb1-C830A-Rev | GCTCCAACCTTCAAGACT**T**CTCTTAAATTAGCCCACT | Introducing SNP mutation into the WT 7637 background (SNP Blb1-7648-830-A) |
| Blb1-C830A-For | AGTGGGCTAATTTAAGAG**A**AGTCTTGAAGGTTGGAGC |  |
| Blb1-G1040A-Rev | GGCACACCACCA**T**TTTTTTTCACAATCTCCTTTCCG | Introducing SNP mutation into the WT 7637 background (SNP Blb1-7648-1040-A) |
| Blb1-G1040A-For | CGGAAAGGAGATTGTGAAAAAAA**A**TGGTGGTGTGCC |  |
| Blb1-C1684T-Rev | CTGCTTTGGAAGACTAC**A**CATGCCACTGCCATACA | Introducing SNP mutation into the WT 7637 background (SNP Blb1-7648-1684-T) |
| Blb1-C1684T-For | TGTATGGCAGTGGCATG**T**GTAGTCTTCCAAAGCAG |  |
| Blb1-T2066G-Rev | ACTTTAACTTCTTCTGATTCATAT**C**TATGTGGTCCAAAGTTATTCCAAC | Introducing SNP mutation into the WT 7637 background (SNP Blb1-7648-2066-G) |
| Blb1-T2066G-For | GTTGGAATAACTTTGGACCACATA**G**ATATGAATCAGAAGAAGTTAAAGT |  |
| Blb1-revert-C830A-Rev | GCTCCAACCTTCAAGACT**G**CTCTTAAATTAGCCCACT | Reverting the SNP mutation present in the 7648 background (WT SNP Blb1-7637-830-C) |
| Blb1-revert-C830A-For | AGTGGGCTAATTTAAGAG**C**AGTCTTGAAGGTTGGAGC |  |
| Blb1-revert-G1040A-Rev | GGCACACCACCA**C**TTTTTTTCACAATCTCCTTTCCG | Reverting the SNP mutation present in the 7648 background (WT SNP Blb1-7637-1040-G) |
| Blb1-revert-G1040A-For | CGGAAAGGAGATTGTGAAAAAAA**G**TGGTGGTGTGCC |  |
| Blb1-revert-C1684T-Rev | CTGCTTTGGAAGACTAC**G**CATGCCACTGCCATACA | Reverting the SNP mutation present in the 7648 background (WT SNP Blb1-7637-1684-C) |
| Blb1-revert-C1684T-For | TGTATGGCAGTGGCATG**C**GTAGTCTTCCAAAGCAG |  |
| Blb1-revert-T2066G-Rev | ACTTTAACTTCTTCTGATTCATAT**A**TATGTGGTCCAAAGTTATTCCAAC | Reverting the SNP mutation present in the 7648 background (WT SNP Blb1-7637-2066-T) |
| Blb1-revert-T2066G-For | GTTGGAATAACTTTGGACCACATA**T**ATATGAATCAGAAGAAGTTAAAGT |  |
| M13-F(-20) | GTAAAACGACGGCCAG | Sequencing primers that are located on the cloning vectors **^(1)^** |
| M13-R(-24) | AGGAAACAGCTATGACCATG |  |
| pDONR201-FOR | TCGCGTTAACGCTAGCATGGATCTC |  |
| pDONR201-REV | GTAACATCAGAGATTTTGAGACAC |  |
| p35S-FOR | AAGGAAGTTCATTTCATTTGGAGAGGA |  |
| t35S-REV | CAACACATGAGCGAAACCCTATAAGAA |  |
| C-mRFP-FOR2 | CCTACAAGACCGACATCAAG |  |
| N-mRFP-REV | TTCAAGTAGTCGGGGATGT |  |
| Cterm-GFP-FOR | ACAACCACTACCTGAGCAC |  |
| Nterm-GFP-REV | CGGACACGCTGAACTTG |  |
| Kana-nptII-F | ATGACTGGGCACAACAGACAATCGGCTGCT | PCR *Kanamycin* *resistance* gene of vector pK7WG2 in OE plants |
| Kana-nptII-R | CGGGTAGCCAACGCTATGTCCTGATAGCGG |  |
| Blb1-F | ATGGCTGAAGCTTTCATTCAAGTTCTG | PCR *Rpi-blb1/RB* gene in OE plants |
| Blb1-R | GGAATGTGAGAAATTTTGTGCCAGTCTTCT |  |
| qPCR-*Blb1*-F | AGGGCTCTTACTTCCCTCAGA | Relative protein expression of *Rpi-blb1/RB* gene in OE plants |
| qPCR-*Blb1*-R | GTGCTGCAATCCCTCTGGTA |  |
| qPCR-*StPR1*-F | TGCCCATGAATATGCCACCA | Relative protein expression of *StPR1* gene in OE plants |
| qPCR-*StPR1*-R | GAGCATTTCAATCTGGCGCA |  |

**^(1)^**	Primer from reference Mei *et al.* (2018). The *Globodera pallida* SPRYSEC effector GpSPRY-414-2 that suppresses plant defenses targets a regulatory component of the dynamic microtubule network. *Frontiers in Plant Science* 9, Article 1019. [doi: 10.3389/fpls.2018.01019]; except for the pDONR201 primers, for which sequences are provided for the first time in the current study.
